# Traditions for Future Cross-National Food Security—Food and Foraging Practices among Different Native Communities in the Western Himalayas

**DOI:** 10.3390/biology11030455

**Published:** 2022-03-16

**Authors:** Shiekh Marifatul Haq, Musheerul Hassan, Hammad Ahmad Jan, Abdullah Ahmed Al-Ghamdi, Khalid Ahmad, Arshad Mehmood Abbasi

**Affiliations:** 1 Department of Botany, University of Kashmir, Srinagar 190006, India; snaryan17@gmail.com; 2 Wildlife Crime Control Division, Wildlife Trust of India, Noida 201301, India; 3 Department of Zoology, Islamia College of Science & Commerce, Srinagar 190001, India; musheer123ni@gmail.com; 4 Department of Botany, University of Buner, Swari 19290, Pakistan; hammadjan@ubuner.edu.pk; 5 Department of Botany and Microbiology, College of Science, King Saud University, Riyadh 1211451, Saudi Arabia; abdaalghamdi@ksu.edu.sa; 6 Department of Environmental Sciences, COMSATS University Islamabad, Abbottabad 22060, Pakistan; khalidahmad@cuiatd.edu.pk; 7 University of Gastronomic Sciences of Pollenzo, 12042 Pollenzo, Italy

**Keywords:** livelihood, climate change, food security, local cuisine, Himalayas

## Abstract

**Simple Summary:**

Biological and cultural history is linked with neglected and underused species (NUS). Wild food plants and animals have been the focus of important investigations carried out around the world in recent decades, and it is seen as a key issue in the fight against hunger and malnutrition. In this study, we assessed the effect of geographical, political, social and economic scenarios of neighboring countries that have led to different food and foraging practices in the erstwhile princely state of Jammu and Kashmir. We recorded 209 species, broadly classified into plants (*n* = 152) and animals (*n* = 57), used by the local people from four biogeographic regions (Kashmir, Jammu, Azad Kashmir and Ladakh) of erstwhile princely state of Jammu and Kashmir. Among wild flora, 139 vascular plants and 13 mushrooms were used by the indigenous communities, whereas in the case of wild fauna, 14 mammals, 22 birds and 21 fishes were used. The Jammu and Kashmir regions showed greater similarity, whereas the least overlap was observed between the Jammu and Ladakh regions. A cross-cultural comparison of wild food resources showed that maximum overlapping was observed in plant resources, and minimum in animals between the four regions of the study area. The usage of different wild foods is also dependent on seasonality. Wild animals and birds are preferred as food in the winter season. However, in the warm months of the year, plants and fish were preferred during the summer season due to their easy accessibility, and they replace the requirement of animals during the warm months of the year. The results of the current study show that the geopolitical scenario in this region has also affected the usage of different animal and plant species for food in the historically unified region of Jammu and Kashmir. This is one of the first comprehensive studies that document the wild food heritage essential for future projects aimed at fostering conservation, environmental sustainability, food security and climate change.

**Abstract:**

Traditional diets exist in all cultures and geographic regions, and they often represent healthy eating options. Traditional culinary preparations have, however, often undergone profound change, even in the isolated Himalayan region. Therefore, we adapted methods to identify traditional plant foraging activities to better understand their significance in food system sustainability, as well as to promote innovative local gastronomies. Information on wild food and foraging practices was gathered from varied ethnic groups such as Kashmiri, Gujjars, Pahari, Dogra, Bakarwal, Balti, Beda and Brokpa through interviews (*n* = 716) and group discussions (*n* = 67) in four bio-geographic regions of the Jammu and Kashmir Himalayas (J&KH). The data were subjected to ordination techniques (Principal Component Analysis) via R software Ver. 4.0.0. We documented 209 food species, of which 73% were plants and 27% animals, used by the inhabitants of four bio-geographic regions of J&KH. The highest number of food plant species was recorded in Indian Kashmir, followed by Jammu, Azad Kashmir and Ladakh (81, 65, 60 and 27 species, respectively), and the maximum number of animal species was reported in Indian Kashmir, followed by Azad Kashmir, Ladakh and Jammu (33, 21, 19 and 17 species, respectively). The Azad Kashmir and Indian Kashmir regions showed greater similarity, whereas the least overlap was observed between Kashmir and Ladakh. The PCA showed considerable variation between different regions, and specific groups of species were more related to one specific region than others. The reported uses of *Abies pindrow, Acacia modesta, Bergenia ciliata, Bergenia stracheyi* and *Juglans regia* among plants, and *Jynx torquilla, Streptopelia orientalis* and *Tadorna ferruginea* among animals, are novel for the gastronomy of this part of the Himalayan region. We also recorded for the first time from this region seven unique food preparations of wild animals. This study documented extensive traditional knowledge on the usage of wild species, and is the first scientific description of wild food species and their vernacular names in the Western Himalayas, Jammu and Kashmir. Our findings can contribute significantly to combating food insecurity by revitalizing and reconsidering the rich bio-cultural food heritage around which local traditional communities have developed their food systems.

## 1. Introduction

The inclusion of indigenous knowledge and ethno-scientific strategies into present frameworks for conservation and sustainable management of natural resources is becoming increasingly important for the development of sustainable food systems [1]. Article 8 (j) of the Convention on Biological Diversity (CBD 2016), clearly endorses Traditional Knowledge (TK) as the base for sustainable development of food systems in a particular region [2]. Bio-cultural refugia [3] act as reservoirs of TK in traditional food systems [4] and have often supported survival during periods of famine [5]. The biological and cultural heritage associated with wild food plants and neglected and underutilized species (NUS) has been at the center of attention of research over the past few decades, and is regarded as a focal issue in battling the problem of hunger and malnutrition [6]. The focus on traditional plant foraging is vital to understanding its role in the sustainability of food systems in remote tribal areas, and in the promotion of novel local gastronomies [7]. TK helps in the discovery of traditional ingredients such as orphan crops, wild crop relatives etc., which can have an important role in combating food insecurity by reviving and reviewing the rich bio-cultural food traditions around which local traditional communities have developed their food systems worldwide [8]. Ethnobiological studies play a vital role in what we call food scouting, i.e., identifying, documenting and disseminating diverse food resources within indigenous communities [9]. Such studies have shown that indigenous communities represent a significant reservoir of disappearing plant and ecological knowledge that needs immediate documentation for developing sustainable food and healthcare systems [10]. Ethno-botanical field surveys can be a viable option for preserving this vital knowledge before it vanishes. 

During the COVID-19 pandemic, amid unstable conditions and issues related to the transport of food and other culinary items, wild food developed new significance as a safety net for food supply and food security. Food security is also jeopardized by climate change. WFPs (Wild-Harvested Food Plants) are vital to the diet of millions of people and contribute to food security, especially in rural and low-income communities, but little is known about their vulnerability to climate change [11]. Changing climatic conditions have already resulted in altered growing conditions for cultivated crops. Climate change will also decrease the per capita land area suitable for food production, making the identification, documentation and dissemination of knowledge about diverse wild food resources even more important [11]. The knowledge of wild animals and plants that grow around us could be essential, specifically in remote rural areas [12]. The erstwhile state of Jammu and Kashmir (hereafter J&K) is divided into four parts, Jammu, Kashmir, Ladakh and Azad Kashmir. The first three parts are controlled by Indian authorities while Azad Kashmir is controlled by the Pakistan government. Additionally, Ladakh region is controlled partly by China and India. In this paper, we hypothesize that the geographical, political, social and economic scenarios of the three neighboring countries (India, China and Pakistan) have led to different food and foraging practices in the erstwhile princely state. This geo-political impact resulting in emigration, religious conversions, cross-ethnic marriages, etc., has affected the usage of different animal and plant species for food in the historically unified region of Jammu and Kashmir, and even an untrained eye can see that while people live in a specific social and economic environment, different geopolitical systems have different uses for wild animals and plants. Traditional wild food knowledge is not only linked to local biodiversity and plant availability, but is also deeply embedded in daily food practices that are highly variable and influenced by a complex combination of socio-cultural factors such as the pervasiveness of industrialized food, food security status/socio-economic conditions, and the importance of cultural identity [10].

In this study, we therefore investigated the effect that linguistic and religious communities have on the consumption of wild plant and animal species in the remote western Himalayas of Jammu and Kashmir.In this study, we tried to answer the questions: (1) to what extent do the geo-political and socio-economic systems of the region impact the local cuisine and uses of wild animals and plants, and (2) why do different groups use wild foods differently in similar regions? Furthermore, this article aims to display comprehensive data about the use of wild animals and plants as food in the Himalayas.

## 2. Materials and Methods

### 2.1. Study Area 

**Figure 1:**
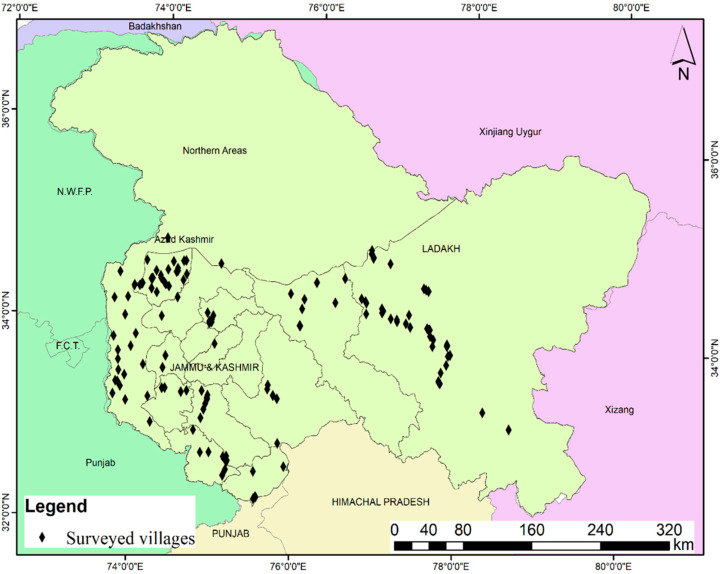
Political map of Jammu and Kashmir (J&K) with neighboring countries, and points showing the surveyed villages in the erstwhile princely state of Jammu and Kashmir. Azad Kashmir region is administrated by Pakistan authorities whereas the Jammu, Kashmir and Ladakh regions are administrated by Indian authorities. The surrounding countries of the region are represented by different colors, with purple representing China and green representing Pakistan while the rest of India is shown in cream.

The present study was carried out in four biogeographic regions (Jammu, Kashmir, Ladakh and Azad Kashmir) (Figure 1), to study the factors leading to food diversity among the local population, differentiated based on religion, language and geography. J&K is located to the north of Himachal Pradesh and Punjab (India) and west of Ladakh. The region is divided into two provinces (Jammu, Kashmir). Ladakh, as mentioned earlier, is ruled by two countries (India, China) and shares its borders with Gilgit–Baltistan (Pakistan) to the northwest, Himachal Pradesh to the west and Tibet to the north. Azad Kashmir (AJK) shares its boundaries to the north with Gilgit–Baltistan (Pakistan), south with Punjab, west with Khyber Pakhtunkhwa and to the east with Kashmir ruled by India. J&K harbors a rich ethnic and cultural diversity. The main ethnic communities are Kashmiri, Gujjar, Pahari, Dogra, Bakarwal, Balti, Beda and Brokpa. The various languages spoken by these ethnic groups are Urdu, Dogri, Kashmiri, Pahari, Gujjari, Hindi, Shina, Balti, Hindko and Ladakhi. 

Kashmiri itself is spoken mainly in the valley (Kashmir), some parts of Jammu, and some parts of Azad Kashmir. Despite the fact that Kashmiri is the most widely spoken language in the region, it is rarely used in education. Dogri, Ladakhi and Pahari are the most widely spoken languages in Jammu, Ladakh and AzadKashmir respectively. Urdu is the only spoken language in all bio-geographic regions (Kashmir, Ladakh, Jammu and Azad Kashmir). Islam and Sikhism are historically the prevailing religions in Kashmir. The Ladakhi people are influenced by the Buddhist faith, and Hinduism is dominant in Jammu (Table 1).

### 2.2. Survey and Data Collection 

**Figure 2:**
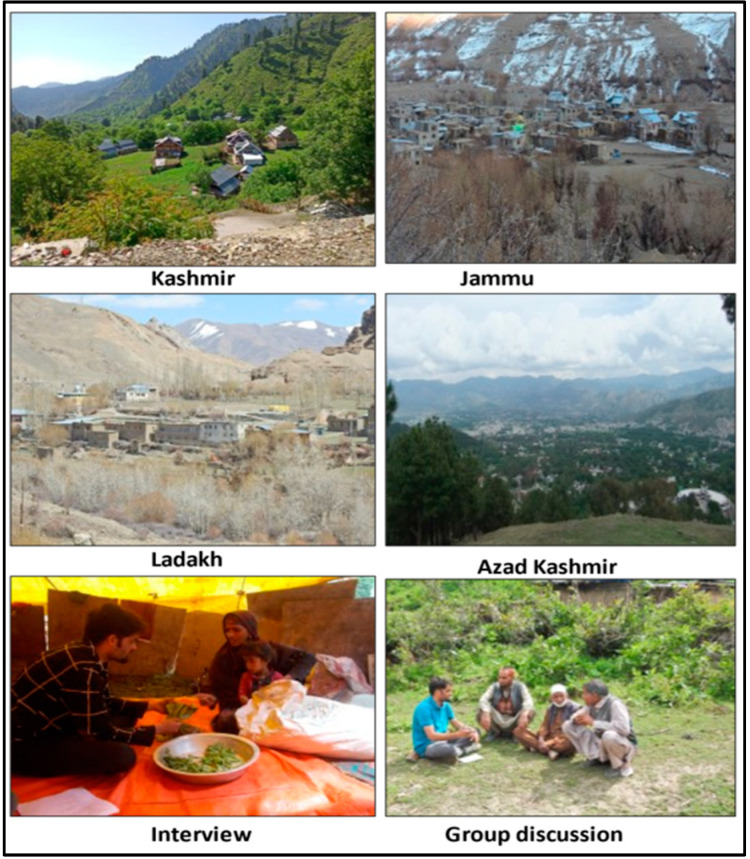
Representative survey sites in different bio-geographical regions for data collection. The photographs were taken by Shiekh Marifatul Haq and Musheer ul Hassan in Jammu, Kashmir and Ladakh, whereas in Azad Kashmir, the photographs were taken by Hammad Ahmad Jan.

The present study was based on the field survey of an area covering 223 villages within the four biogeographic regions (Jammu (*n* = 58), Kashmir (*n* = 62), Ladakh (*n* = 54) and Azad Kashmir (*n* = 49)) (Table 1, Figure 1 and Figure 2). Representative villages were visited during 2019 and 2021. Members of different ethnic groups like Pahari, Gujjar, Kashmiri, Dogra, Bakarwal, Beda, Brokpa and Balti, whofollow different faiths like Islam, Hinduism, Sikhism and Buddhism, were interviewed across the study area. A total of 952 respondents were interviewed, among them 729 men and 223 women. The representative sites were visited several times (37 visits) during the survey period. The sampling was carried out in all four seasons of the year i.e., spring (March–May), summer (June–August), autumn (September–November) and winter (November–February) to document the seasonal use of wild plants and animals across the regions. The stratified random sampling method was used for documentation, which included interviews (*n* = 716), followed by group discussions (*n* = 67) [13]. Prior informed consent was taken from the participants, and all the interviews were conducted in local languages using translator services if required. The ethnicity of the participants and the language information given are not disclosed, based on mutual agreement, as stipulated under the Nagoya Protocol. The Code of Ethics of the International Society of Ethnobiology was followed [14]. Information about the plant/animal local names, parts used, valuable key species, collection season and availability status, preferred species and demographic profile of the study participants were documented. The interviews were conducted across different age groups i.e., young (26.05%), middle (31.89%) and old (42.12%), and gender groups, i.e., men (77%) and women (23%). Traditional knowledge was collected from different occupational groups of the region [15,16] (Table 1). The most important knowledge holders were elderly people, and while most respondents were unschooled (65.65%), 21.11% had benefited from primary education, 12.18% from secondary-level education and only 1.05% from higher education. Ten languages (Urdu, Gujjari, Pahari, Kashmiri, Balti, Hindko, Kitwari, Shina, Dogri and Ladakhi) were documented across the study area, and among them, Urdu and Kashmiri were the only commonly spoken languages (Table 1).

One knowledgeable respondent from each research site was asked to accompany the researchers to collect plant specimens for verification and herbarium preparation. Murthi [17] and Menon [18] were used for identification, and respondents were shown pictures or live plants for local names. In addition, whenever there was any discrepancy in names, a group discussion was used as a methodology to remove any bias. The collected specimens were further checked for proper identification with the help of taxonomists at the Centre of Biodiversity and Taxonomy, University of Kashmir, Srinagar (J&K), while the specimens collected from Azad Kashmir were identified by Dr. ZahidUllah, Assistant Professor, Department of Botany, University of Swat, and Dr. Sher Wali Assistant Professor, Department of Botany, Islamia College, Peshawar. The online “Flora of Pakistan” was used to confirm the correct nomenclature (http://www.tropicos.org/project/pakistan accessed on 12 April 2021). The collected specimens were authenticated at KASH herbarium and at the herbarium of the Department of Botany, Islamia College, Peshawar.

### 2.3. Data Analysis

Associational analyses among different regions and plant/animal compositions were carried out using Principal Component Analysis (PCA) [13] to find hypothetical variables (components) that accounted for as much variance in our multidimensional data as possible. For that, we used a matrix of presence/absence of animal and plant species in each of the four regions studied and calculated the singular value decomposition of the (centered and possibly scaled) data matrix. PCA was performed using the Software R Studio 4.0.1. With PCA, we were able to investigate how each, or a set of, species are related to each region evaluated. Cluster analysis was carried out to find out how the diversity is related to different ethnic groups using PAST software ver.3.14. This method allows characterization from the individual samples and then combines these into groups, in terms of their similarity. The presence or absence of each species was determined based on the species’ use by the specific group in the region. The Venn diagram was prepared using Bioinformatics & Evolutionary Genomics software (available at http://bioinformatics.psb.ugent.be/webtools/Venn/ accessed on 12 May 2021).

## 3. Results

### 3.1. Wild Food Domain

**Figure 3:**
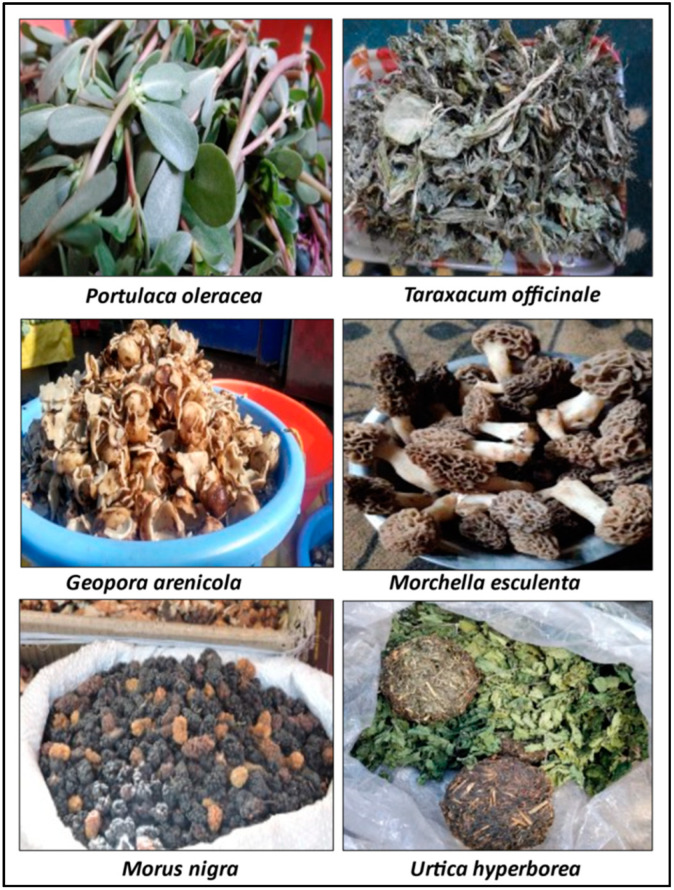
Representative species used as wild food (plants). The photographs were taken by Shiekh Marifatul Haq and Musheer ul Hassan.

**Figure 4:**
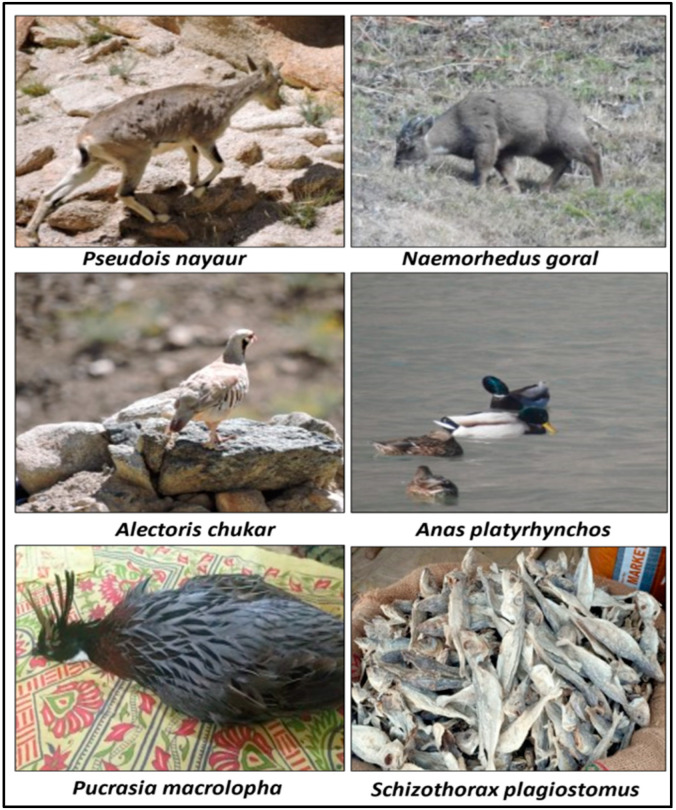
Representative ethno-wild food (animals) used by indigenous people. The photographs were taken by Shiekh Marifatul Haq and Musheer ul Hassan.

**Figure 5:**
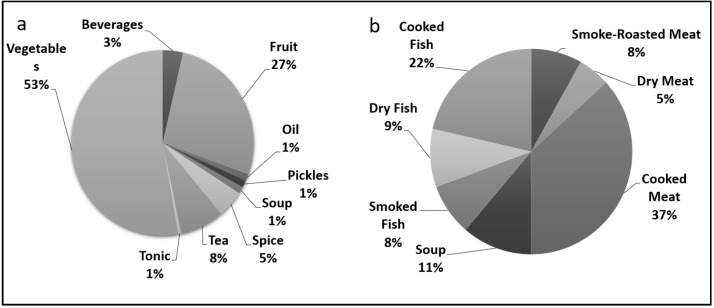
Consumption percentage of food preparation (**a**) plants (**b**) animals, birds and fish in the erstwhile state of J&K.

We recorded 209 species, broadly classified into plants (*n* = 152; 139 vascular plants, 13 mushrooms) and animals (*n* = 57; with 14 mammals, 22 birds and 21 fish) used by the local people from four biogeographic regions (Table S1). (Figure 3 and Figure 4). The local population consumed more than half (53%) of the plants as vegetables, followed by fruits (27%), and tea (8%) (Figure 5a). Food preparation has been studied by various researchers e.g., Majeed et al. [19], Manduzai et al. [20] from Pakistan Himalayas; Boesi [21] from Tibetan communities; de Medeiros et al. [22] from Brazil; Stryamets et al. [1] from Ukrainian and Romanian Bukovina. Here we report seven unique types of food preparation from different animal species in the region (Figure 5b). Cooked meat (37%) was the most popular preparation, followed by cooked fish (22%),soup (11%) and other preparations with less than 10% contribution are shown in Figure 5b. The prevalent usage of plants is seen as a result of diverse vegetation types (subtropical to alpine) across a wide elevational gradient [23], similar to other ethnobiological studies [24,25,26].

**Figure 6:**
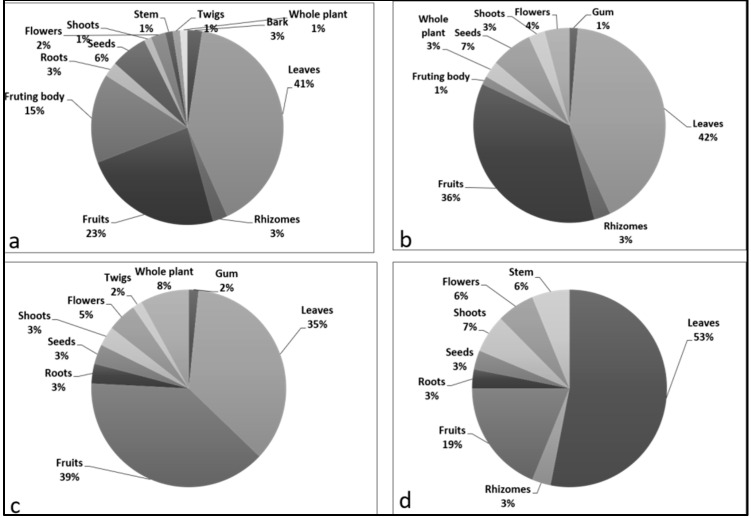
Comparative percentage of different plant parts used in (**a**) Kashmir (**b**) Jammu (**c**) Azad Kashmir (**d**) Ladakh region.

In Kashmir (valley of Kashmir), 81 plant and 33 animal species were documented as wild food, including mammals (*n* = 5), birds (*n* = 10), fish (*n* = 18). Among plants, leaves were the most used part (41%), followed by fruits (23%) and fruiting body (15%) (Figure 6a). The dominant plant families used were Polygonaceae (10%), Rosaceae (10%), followed by Asteraceae (6%), Lamiaceae (4%). This dominance of Polygonaceae, Rosaceae, Asteraceae might be due to suitable habitat, and favorable environmental conditions for the growth of the species belonging to these families. Traditional uses of these species are well recognized by the local inhabitants [13,27].

In animals, meat (43%) was found to be the dominantly consumed part followed by coagulated protein (24%) from fish, and gizzard (7%) from birds. Ishtiyak et al. and Altaf et al. [28,29] reported the same from Punjab, Pakistan. Among animals, Cyprinidae (40%) was the dominant family, followed by Columbidae (9%), and Phasianidae (9%) (Table S1).

In Jammu, we found 65 plant species and 17 animal species employed as wild food. Out of 17 animal species, 8 species were mammals, 2 species were birds, and 7 were fish. Contrary to Kashmir, Jammu had less diversity in wild food usage. This can be explained by the fact that Jammu is nearer to the agricultural state of Punjab, and the population mostly relies on cultivated food. Jammu also has a majority Hindu population who are vegetarian. Leaves were used as primary food (42%), followed by fruits (36%), and seeds (7%), similar to Kashmir (Figure 6b). Leaf greens and aerial parts were regarded as safe and sustainable [30,31]. Rosaceae (10%) and Polygonaceae (10%) were dominant families. The most favored animal parts were meat (30%), followed by coagulated protein (12%), trotters (11%), heart (11%), lungs (11%), and kidney (11%). Animals belonged to a variety of families; however, the dominant families recorded were Cyprinidae (29%) and Bovidae (29%), followed by Cervidae (12%) and Phasianidae (12%). 

In Azad Kashmir, 60 plant species and 21 animal species were used as wild foods. Amjad et al. [32] also reported the use of animals (*n* = 4), birds (*n* = 6) and fish (*n* = 11) from Pakistan. The use of plant species was lower than Kashmir and Jammu, but animal usage was higher than Jammu and lower than Kashmir. The inhabitants of Azad Kashmir are mostly Muslim, and hence consume more animals than the Jammu population. However, their population is smaller than that in Indian Kashmir, which is also a Muslim majority area. Fruits (39%) were the most used plant parts, followed by leaves (35%) (Figure 6c) with dominant families Asteraceae (12%), followed by Rosaceae (7%) and Lamiaceae (5%). In comparison to cultivated species, wild fruits were mostly consumed raw, being known to contain more fiber, higher vitamin concentrations, and a greater range of secondary metabolites [33]. The dominance of Asteraceae was similar in other studies [32,34,35]. Among animals, the most used parts were meat (40%), coagulated protein (21%) and kidney (8%). Cyprinidae (39%) was the leading family, followed by Columbidae (17%) and Salmonidae (11%).

People in Ladakh, with its high altitude and harsh climatic conditions, used only 27 plant species and 19 animal species (4 mammals, 6 birds and 11 fish) as wild foods. The study showed that the use of wild flora and fauna was more limited due to the harsh dry arid climate. We also observed that food usage was influenced by China due to historic trade via the Silk Route, which resulted in the influx of a variety of food traditions from China into the region, and later got blended into the local food culture. Furthermore, it is important to mention that present-day Ladakh was once ruled by China, and it was the Mughal Empire that annexed it with the Kashmir province. However, the deep-rooted Chinese culture is still clearly visible in the region. Leaves (53%), followed by fruits (19%), were the main parts of the plant used frequently (Figure 6d). Boesi [21] and Pala et al. [36] also reported leaves as the most utilized part from the Eastern Himalayas. Polygonaceae were the dominant (22%) plant family, followed by Asteraceae (11%), Rosaceae (7%) and Apiaceae (7%). Meat (36%) was the most used animal part, followed by gizzard (15%), spleen (8%) and kidney (8%). Bovidae (26%) was the dominant family followed by Phasianidae (21%) and Columbidae (16%). Haq et al. [13] also reported the dominance of Bovidae. Meat was praised as nutritious, and used mostly in Kashmir (43%) and least in Ladakh (36%) (Table S1). The regional variation in species use patterns can be attributed to the different regions’ geopolitical and socioeconomic systems (Table 1). It is also worth noting that religious affiliations have an impact on every region’s usage pattern. Hindu and Buddhist religious groups are reluctant to hunt wild animals for food, but the Muslim community does so. Similarly, while Muslim scholars in Kashmir prohibit using *Allium semenovii* before prayers, other religious groups do not. All these factors influence local cuisine and the use of wild animals and plants in the study area.

### 3.2. Seasonal Usage of Wild Foods

The usage of different wild foods was dependent on seasonality. Wild animals and birds were preferred food because of their availability in the winter season, when wild plants were not in abundance. Wild animals and birds were also preferred as food in cold, harsh weather because of their high nutrient and fat content. However, in the warm months of the year, plants were preferred as they were easily available. In addition, fish were also preferred during the summer season due to their easy availability, and they replaced the requirement of animals during the warm months of the year.

### 3.3. Novel Species Having Gastronomic Application 

In the present study, some documented food species had never been reported from these biogeographic regions. The root bark from *Abies pindrow*, the rhizome of *Bergeniaciliata, B. stracheyi, Rheum webbianum* and *Acorus calamus,* and leaves from *Cichorium intybus*, *Origanum vulgare* were used as herbal tea. Young leaves of *Vitis jacquemontii* were used as salad. Male inflorescences of *Juglans regia* were used as vegetables, and fruits of *Ziziphus jujube*, *Ziziphus nummularia* were eaten. The meat of *Jynx torquilla, Streptopelia orientalis and Tadorna ferruginea* were used as food in the Kashmir region. Similarly, leaves from *Cichorium intybus* and *Origanum vulgare*, and the rhizome of *Bergenia ciliata* and *B. stracheyi,* were used for making herbal tea in the Jammu region. In Azad Kashmir, young leaves of *Conyza canadensis* were cooked with rice. *Acacia modesta* gum was used as honey replacement. *Arnebia euchroma* roots were used as spice, and leaves taken with tea to enhance flavor. Tender leaves and young shoots of *Leucas cephalotes* were cooked as vegetable, as were leaves and young plants of *Onopordum acanthium*. The ripe fruits of *Sambucus wightiana* were eaten raw. The meat of *Capra falconeri, Muntiacus muntjak, Hrundo rustica, Oenanthe oenanthe, Passer domesticus, Streptopelia decaocto* and *Streptopelia tranquebrica* was used as food. *Columba livia* was used to prepare soup. The meat of *Barbus sarana, Catla catla, Cirrhinus cirrhosis, Labeo calbasu, Labeo dero, Oncorhynchus mykiss, Puntius ticto, Salmo truttafario, Schizothorax plagiostomus, Triplophysa kashmirensis* and *Glyptothorax kashmiriens* was cooked. *Prunus armeniaca* was sun dried, stored and used in harsh winters as a vegetable in Ladakh. 

### 3.4. Wild Food Usage across Regions 

**Figure 7:**
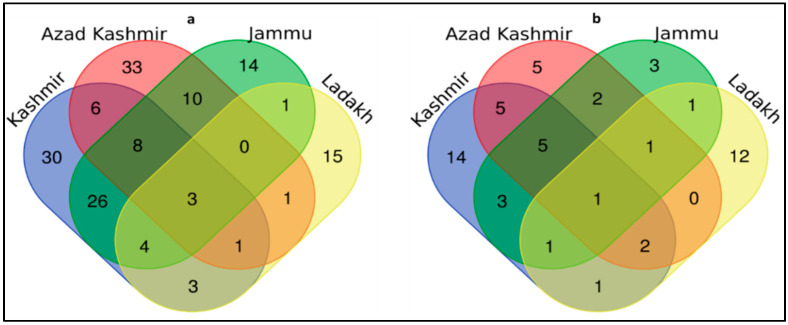
Venn diagram (**a**) representing unique and common plant species (**b**) representing unique and common animal species among different regions.

The Venn diagram (Figure 7a) shows that the maximum use of plants was reported from Azad Kashmir, while Jammu region reported the minimum. The J&K region showed greater similarity, whereas the least overlap was observed between Jammu and Ladakh. A cross-cultural comparison of plant resources showed that 63 plants were overlapping between the four regions of the study area. The reason for the widespread use of plants in Azad Kashmir lies in the fact that the region is mountainous, rural and less developed, and thus local people are more dependent on wild plants when compared to the Jammu region, which is less hilly and more developed than other parts of the state. Also, because the Jammu region is well connected to the rest of the country, the easy availability of cultivated food reduces the region’s reliance on wild food.

**Figure 8:**
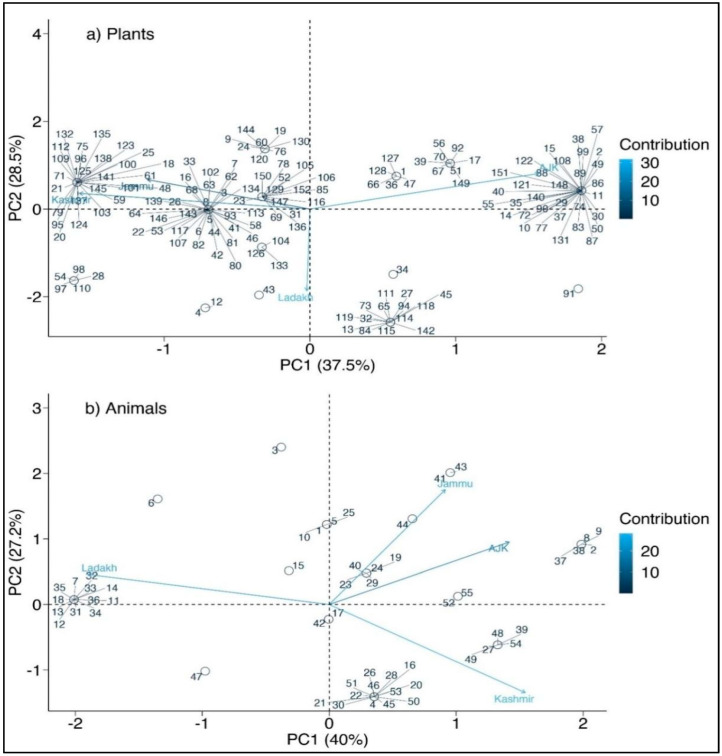
PCA diagram (**a**) representing clustering of plant species (**b**)representing clustering of animal species among different regions. The complete name of species as per the serial numbering in Table S1.

The highest use of wild fauna was reported in the Kashmir region, followed by Ladakh, while the Jammu region reported a minimal number of animal uses. Azad Kashmir and Kashmir regions showed greater similarity, whereas the least overlap was observed between Kashmir and Ladakh. A cross-cultural comparison of animal resources showed that 22 animals were overlapping between the four regions of the study area (Figure 7b). The maximum use of wild fauna in the Kashmir region occurs because, in the winter season, the locals prefer to eat wild meat for energy, and people feel that wild meat is free of cost. The greater similarity between the Azad Kashmir and Kashmir regions is due to closer geographic, cultural and religious resemblance between the two regions. Similarly, the PCA showed considerable variation in different regions (Figure 8a,b). Based on species presence/absence discovered in the case of plants, PC1 and PC2 described 66% of species distribution in the biplot, in which J&K region species are grouped on one side of the PCA and Azad Kashmir forms a separate cluster on the other side (Figure 8a). However, in the case of animals, the PC1 and PC2 explained 67.2% of the species distribution, with the Ladakh region forming discrete clusters from the rest of the regions (Figure 8b). Other studies, e.g., [19,20,37] from Pakistan, Stryamets et al. [1] from Ukraine, also reported the cross-cultural use of wild foods.

### 3.5. Wild Plants as Vegetables

Throughout the entire study area, the use of herbs as vegetables was common. Leaves were the most favored part. Women were the leading source of knowledge about nutritional aspects of medicinal and edible species, given that they mostly collected comestible and medicinal species, and were directly involved in household and food preparations [38,39,40,41,42,43,44].

The fruits of *Phylanthus emblica, Solanum nigrum* and *Trichosanthes cucumerina* were cooked as vegetables. Similarly, the leaves of *Malva neglecta, Medicago polymorpha, Oxalis corniculate, Oxyria digyna, Phytolacca acinose, Plantago depressa, Plantago lanceolata, Plantago major, Viola odorata, Taraxacum officinale, Rheum emodi, Rheum spiciforme* and *Rheum webbianum* were also consumed. Young twigs of *Dryopteris stewartii* were boiled, dried and used as vegetable during the winter season. A number of reports from the Himalayas confirm this use of wild plants as a vegetable [21,45,46,47]. People in Kashmir, Jammu province and Azad Kashmir collected especially the young and fresh leaves of *Allium humile, Amaranthus viridis, Berberis aristata, Cardamine hirsuta* and *Nasturtium officinale* as vegetables [48].

### 3.6. Wild Plants as Fruits

Fruits were mostly used raw, and sometimes dried [49]. In this study, fruits like *Hippophae rhamnoides, Prunus armeniaca, Prunus domestica* and *Vitis jacquemontii* were mostly used in Ladakh, where the local inhabitants would sundry and consume them in harsh winter. *Punica granatum* was used in Azad Kashmir and Kashmir. Local inhabitants used the raw fruit, and sometimes the juice. Similarly, *Pyrus pashia, Zanthoxylum armatum* and *Ziziphus jujube* were used in Jammu and consumed when fully ripe. The use of wild fruits is reported throughout the globe. Ojelel et al. [50] reported the use of wild fruits from Uganda; Mahapatra and Panda [51] reported the use of wild fruits from eastern India; and Khan et al. [52] reported the same use from Swat Pakistan. 

### 3.7. Wild Plants as Spices and Oil

Kashmir is known for its cuisine, especially in Wazwaan and other food recipes. The most common wild plant materials used were the leaves of *Mentha arvensis.* Similarly, *Sesamum orientale* was used to garnish local bread and cakes. In Jammu, dried flowers of *Micromeria biflora* were used by various tribal people. Most of the usage was as flavoring agents in curries and soups. *Nepeta floccosa* was documented from Ladakh, and the aromatic dried leaves and shoots were used to add flavor to local dishes. Our results agreed with Aryal et al. [45], who reported the use of wild plants for cuisine in the western Himalayas; and Bhatia et al. [33], who reported the use of wild plants as cuisine in Udhampur—Jammu.

### 3.8. Wild Animals as Bushmeat

Meat and internal organs of *Boselaphus tragocamelus, Capra falconeri, Capra sibirica, Cervus elaphus hangul, Hemitragus jemlahicus, Lepus oiostolus, Marmota himalayana, Moschus moschiferus, Muntiacus muntjac, Naemorhedus goral, Ovis ammon, Ovis aries vignei, Procapra picticaudata* and *Pseudois nayaur* were often eaten locally. Similarly, Altaf et al. [29] reported the use of various animal species from Pakistan. We found that the presence and usage of wild fauna was similar in the whole study area. In Kashmir, *Moschus moschiferus* was the most hunted animal species, both for meat and musk. Similarly, *Capra falconeri* was killed in Azad Kashmir, *Boselaphus tragocamelus* was poached in Jammu, and *Capra sibirica* in Ladakh. Similar exploitation was found by Mahawar and Jaroli [53]. Local Ladakhi people also hunted wild animals like *Alectoris chukar* and *Pseudois nayaur* (blue sheep), and dried the meat for the harsh winter months. On religious grounds, only certain species of birds and animals were used by Muslims as they follow Islamic teachings, especially in multi-religious areas. Muslim groups typically hunted some bird and mammal species, but Buddhists also gathered meat from animals killed by predators such as snow leopards, bears and wolves, or through natural death [13].Hindu people are reluctant to use species like *Boselaphus tragocamelus* due to their faith orientation.

### 3.9. Birds as Food

The meat, gizzard and eggs of *Alectoris chukar*, *Anas acuta*, *Columba livia,* etc, were taken as food (Table S1). Similarly, Mahawar et al. [53] reported the usage of birds like *Columba* sp. from Rajasthan, India; Powell et al. [54] also reported the use of wild birds from India. 

### 3.10. Fishes as Food

The meat of *Barbus sarana*, *Catla catla*, etc, was used as food (Table S1). The documented *Schiziothorax* species are cold freshwater fish native to J&K [55]. These species are known for their taste, and are preferred over *Catla catla* and *Labeo calbasu*. *Schizothorax plagiostomus* is also found in Azad Kashmir, and *Schizothorax labiatus* in Ladakh. Other famous fish species from Azad Kashmir include *Triplophysa kashmirensis* and *Glyptothorax kashmiriensis.* Our present study is in accordance with [29,56]. 

### 3.11. Cross-Cultural Analysis 

**Figure 9:**
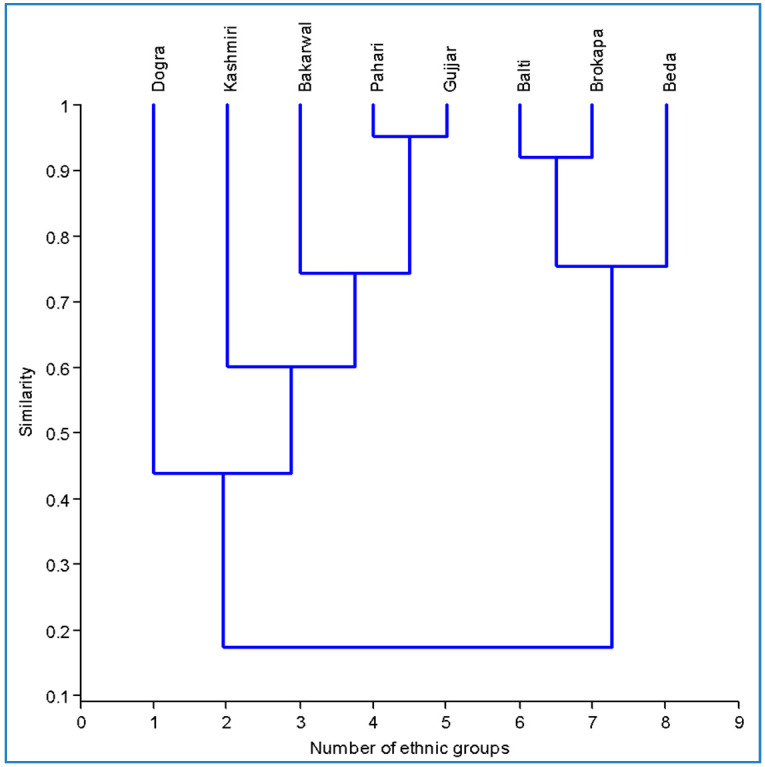
Dendrogram representing clustering of investigated ethnic groups among different regions.

Local inhabitants living at or around higher altitudes of the Himalayas harbor ancient cultural practices in utilizing wild edible plant species [47,57,58]. In this study, we analyzed the utilization of wild foods based on preference, and seasonal and cultural availability. This was further supported by cluster analysis that grouped the eight ethnic groups into two primary clusters based on similarity in wild food use (Figure 9). The ethnic groups grouped together in the dendrogram were more similar in species utilization and lived in close proximity to one another. The Dogra and Kashmiri ethnic groupings were separated on the dendrogram’s extreme left, establishing independent branches with the least degree of similarity. This can be ascribed to the variance in their language and religion, besides other social differences. On the other hand, Gujjar and Pahari ethnic groups formed the most comparable cluster, which was due to them having the same socio-economic status (Table 1), and the fact that both ethnic groups were seen to live close to each other, and share common environment and natural resources across the region. At cut level 2, the greatest resemblance was found between Balti and Brokpa. Both ethnic groups are unique to Ladakh, and hence share the same geography and livelihood practices like cattle rearing and horticulture. The use of *Malva neglecta, Taraxacu mofficinale, Stellaria media* and *Thymus linearis* was found to be common in Gujjar and Pahari diets, especially as a salad or soup, whereas *Berberis lycium, Ficus carica, Fragaria nubicola, Rubus fruticosus* and *Rubus ellipticus* were the most consumed fruits (Table S1). Both ethnic groups (Gujjar and Pahari) have a strong cultural belief that wild plants provide nutrients [59]. Maundu et al. [60] reported that wild varieties are rich in essential micronutrients, vitamins and minerals. Balti and Brokapa consider *Urtica hyperborea* soup as potential food that is rich in nutrients, while *Prunus armeniaca* is cooked as a vegetable. People following Islam use this species during Ramadan frequently, believing it as a rich source of energy. The gathering of wild edible mushrooms is a common and traditional practice, especially in Azad Kashmir and Kashmir. People enjoy their meals by including several species of mushrooms such as *Morchella esculenta, Morchella vulgaris, Geopora arenicola* and *Flammulina velutipes* in their dishes. These mushrooms also hold good economic value and are often sold [61].

### 3.12. Wild Food in COVID-19 Pandemic

A Food and Agriculture Organization (FAO) study reports that approximately 1 billion people worldwide use wild plants as a source of their diet [61,62]. The COVID-19 epidemic is expected to significantly expand the use of wild plants, e.g., as herbal ingredients in traditional Chinese Medicine (TCM) formulations as well as other herbal products around the world. In the study area, people from all four regions boiled wild plants and prepared “kadda” to protect against and cure symptoms of COVID-19. Some of the most commonly used plant species included *Arnebia bentami, Adiantum capillus-veneris, Origanum vulgare, Saussurea costus* and *Taraxacum officinale,* often used to treat respiratory ailments [42]. In our study, it was found that some medicinal herbs such as *Datura stramonium (Datur-bool), Rheum webbianum (Pambchalan), Artemisia absinthium (Tethwan), Origanum vulgare (Wan baber)* and *Prunella vulgaris (Kale voth)* were used as immunity boosters during COVID-19. *Prunella vulgaris (Kale voth)*, *Cichorium intybus* and *Salix alba* (*Yeed*) were mixed and boiled to make herbal tea. *Arnebia benthamii (Kahzaban) and Viola odorata* (Banafsha) were boiled together to make a drink (*Sharbat).* This drink was also given to patients for relief from chest and throat problems, especially during the COVID-19 pandemic to enhance immunity. 

### 3.13. Food Security and Wild Foods

In the study area, wild food plants have remained an essential component of the local food basket. We visited pastures in the upper elevations of valleys known locally as “Gurez” in Bandipore, “Keran” in Kupwara, “Chinab" in Ramban-Banihal, and “Neelam valley" in Azad Kashmir during the field survey. According to another study carried out in Chitral, Northwest Pakistan [10], the summer meadows are considered reservoirs of several important food and medicinal plant species. We also discovered other plants being sold in markets throughout the study, including *Taraxacum officinale, Morchella esculenta* L, *Gyromitra esculenta* (Pers.) Fr. and *Rheum webbianum,* which are popular wild plants in the studied region. These wild species play an important role in regional diets, especially when there is a food shortage due to drought or disease. People with limited income eat wild food species because they are plentiful and easy to obtain. However, there are certain factors that are becoming potential threats to these natural gifted species, and they include heavy livestock grazing and associated disturbance, habitat fragmentation, unwise development and poaching. The World Food Program reports that 130 million more people are hungry due to drought, and about 135 million are now at risk of extreme starvation [63,64]. Several intense hotspots of starvation have also arisen. As stated by the UN, about 45 million citizens, primarily in Asia and Sub-Saharan Africa, were in acute food shortage between February and June 2020 [65]. While some of these countries manage their food supply portfolios well, others face real environmental limitations in producing more food at home [66]. 

### 3.14. Climate Change and Wild Foods 

The rising difference between food supply and the population is an important challenge for human survival in developing countries. In these countries, food malnutrition occurs due to less intake of fruits and vegetables. Thus, with higher cost or market value, the people of developing countries—mostly children—are unable to buy them and thereby become prone to mineral and vitamin deficiencies [67]. A different approach to food safety is to cultivate and use wild plants to mitigate food shortage and malnutrition. Wild edible plants are cheap, high in antioxidants, vitamins, fiber and minerals. Low transmission of traditional knowledge among younger generations and changing food habits are the leading cause for loss of traditional and beneficial knowledge among local inhabitants [68,69,70,71]. The involvement of more researchers is the need of the hour, transforming traditional aspects into scientifically proven conclusions. In our study, we found that there are many species, such as *Portulaca oleracea, Amaranthus viridis, Capsella bursa-pastoris, Duchesnea indica, Fragaria nubicola, Mentha arvensis, Taraxacum officinale* and *Rubus ellipticus*, which have great nutritional value and these species grow under changing climatic conditions as weeds. However, most of these species are neglected and underutilized, and are mostly used only by a few ethnic communities. Hence, if these plants are brought into the mainstream, they will play an important role in ensuring food sustainability and security despite future climate change. Amid changing climatic conditions, cultivated crops have become more prone to disease and other drastic climatic conditions. So, in place of these, wild varieties can be grown because they are resistant to pestsand changing climatic conditions, literally becoming a source of food security for rural and tribal communities. Hence, to bridge the gap created by climate change, food insecurity and population explosion, it makes perfect sense to switch to wild edible foods found locally.

### 3.15. Wild Foods as Livelihood Generation 

Shepherds feed their livestock in the grasslands of Ladakh and forests of Kashmir, Jammu and Azad Kashmir, and know much about wild plants with market value. Species like *Aconitum heterophyllum*, *Bergenia ciliata*, *Dioscorea deltoidea*, *Amaranthus viridis*, *Geopora arenicola*, *Rheum webbianum*, *Taraxacum officinale*, *Saussureacostus*, *Morchella esculenta*, *Mentha arvensis*, *Urtica hyperborea* and *Viola biflora* have been considered economically important plant species that can generate income for the local population. Farmers are managing their lands by growing wild plant species that provide them economic sustainment under agroforestry regimes, and these include *Juglans nigra*, *Pyrus pashia*, *Punica granatum*, *Emblica officinalis*, *Hippophae rhamnoides*, *Vitis jacquemontii*, *Morus alba*, *Morus nigra*, *Zanthoxylum aromatum* and *Ziziphus nummularia*. This demonstrates that such species management and acquisition of economic benefits can inspire the local people’s interest in wild plant conservation and maintenance (Table 1). 

## 4. Conclusions

This study has gathered a lot of traditional knowledge on the usage of wild species, and contains the first scientific description of wild food species and their vernacular names in the Western Himalayas, Jammu and Kashmir. We recorded 209 species, broadly classified into plants (*n* = 152; 139 vascular plants, 13 mushrooms) and animals (*n* = 57; with 14 mammals, 22 birds and 21 fish) from four biogeographic regions used in traditional feeding systems by the local people. The local population consumed more than half (53%) of the plants as vegetables, followed by fruits (27%), and tea (8%). A surprising quantity of wild food plants as well as wild plants were utilized as snacks, possibly indicating the pastoral lifestyle these people have been leading for years. The most commonly consumed plant components in the research area were its aerial parts and fruits. A wide range of edible wild food species with multiple uses as fruits, vegetables, spices and processed food products are being utilized by the inhabitants of Jammu & Kashmir Himalayas. These food resources have long remained underexploited due to lack of awareness. Cultivating wild food plant species may provide people with the opportunity to supplement their household income, particularly in poor rural areas. Popularizing these species among farmers with due market support, specifically fruits and vegetables, could ensure their profitable farming and effect significant on-farm conservation of valuable germplasm. Moreover, large areas of degraded forest lands and other wastelands areas in Jammu and Kashmir could possibly be reclaimed by planting wild species, which are easier to grow even under adverse soil and climatic conditions. At the same time, these plantations would also benefit wild fauna by providing them with suitable habitat and food. There is a need to develop a value chain from production/processing to marketing/consumption of these underutilized wild food plants to obtain satisfactory economic returns. At the same time, the role of agricultural scientists is to provide technical know-how and find a suitable place for these species in the changing farming patterns due to inevitable climate change, thereby saving valuable wild food resources from extinction.

## Figures and Tables

**Table 1:** Demographic status of the respondents from the study area.

Demographic Features	Number	Bio-Geographic Regions													
		Jammu (JAM)				Kashmir (KAS)			Ladakh (LAD)			Azad Kashmir (AJK)			
**Respondents**	952	259 (27.20%)				224 (23.52%)			199 (20.90%)			270 (28.36%)			
**Approx number of inhabitants**		3 million				4 million			0.25 million			4 million			
**Villages visited**	223	58 (26.00%)				62 (27.8%)			54 (24.21%)			49 (21.97%)			
**Vegetation type**		Subtropical, moist temperate, subalpine				Dry temperate subalpine to alpine			Alpine			Sub-tropical, moist temperate, subalpine to alpine			
**Ethnic groups**		Pahari	Bakarwal	Gujjar	Dogra	Kashmiri	Pahari	Bakarwal	Balti	Brokpa	Beda	Kashmiri	Pahari	Bakarwal	Gujjar
**Language**		Pahari	Gujjari	Gujjari	Dogri	Kashmiri	Pahari	Gujjari	Balti	Brokpake	Bhodi	Kashmiri	Pahari	Gujjari	Gujjari
**Socio-economic status**		Horticulture and Cattle rearing	Pastoralism	Horticulture and Cattle rearing	Horticulture and Cattle rearing	Horticulture and Cattle rearing	Horticulture and Cattle rearing	Pastoralism	Cattle rearing and Horticulture	Cattle rearing, Horticulture and Wage labour	Agriculture and Music	Horticulture and Cattle rearing	Horticultureand Cattle rearing	Pastoralism	Horticulture and Cattle rearing
**Religion**		Hinduism, Islam, Sikhism				Islam, Sikhism			Buddhism, Islam			Islam			
**Education**															
*Unschooled*	625	162 (17.01%)				111 (11.65%)			167 (17.54%)			185 (19.43%)			
*Primary education*	201	63 (6.61%)				74 (7.77%)			21 (2.20%)			43 (4.51%)			
*Secondary education*	116	30 (3.15%)				36 (3.78%)			19 (1.99%)			31 (3.25%)			
*Higher education*	10	4 (0.42%)				3 (0.31%)			1 (0.10%)			2 (0.21%)			
**Age range**															
*Young (18–26)*	248	64 (6.72%)				66 (6.93%)			45 (4.72%)			73 (7.66%)			
*Middle (27–55)*	303	86 (9.03%)				75 (7.87%)			51 (5.35%)			91 (9.55%)			
*Old (56–75+)*	401	109 (11.44%)				83 (8.71%)			103 (10.81%)			106 (11.13%)			
**Profession**															
*Daily wage laborers*	118	24 (2.52%)				30 (3.15%)			29 (3.04%)			35 (3.67%)			
*Herders*	113	28 (2.94%)				33 (3.46%)			25 (2.62%)			27 (2.83%)			
*Farmers*	203	63 (6.61%)				48 (5.04%)			40 (4.20%)			52 (5.46%)			
*Shopkeepers*	232	66 (6.93%)				45 (4.72%)			46 (4.83%)			75 (7.87%)			
*Skilled/semi-skilled laborers*	199	53 (5.56%)				47 (4.93%)			44 (4.62%)			55 (5.77%)			
*Houewives*	87	25				21 (2.20%)			15 (1.57%)			26 (2.73%)			
**Gender**															
*Male*	729	198				172 (18.06%)			154 (16.17%)			205 (21.53%)			
*Female*	223	61				52 (5.46%)			45 (4.72%)			65 (6.82%)			

## Data Availability

All the data obtained during the study are included in this article and the supplementary material.

## Supplementary Materials

The following supporting information can be downloaded at: https://www.mdpi.com/article/10.3390/biology11030455/s1, Table S1: Gastronomic usage of local flora and fauna in different regions of study area.
