# Supplementary material for: Traditions for Future Cross-National Food Security—Food and Foraging Practices among Different Native Communities in the Western Himalayas
Source: Biology (Basel). 2022 Mar 16;11(3):455. doi: 10.3390/biology11030455 (PMC8944997; doi:10.3390/biology11030455)
Supplement: Supplementary file 1 [file biology-11-00455-s001.zip › biology-1553935-supplementary.pdf]

Table S1. Gastronomic usage of local flora and fauna in different regions of study area

S.No.	Species (Family) (Voucher number)	Vernacular name	Part used	Usage	Bio-geographic regions				Ethnic groups	Use value	Citation
					KAS	JAM	LAD	AJK			
Plants											
1	*Abies pindrow* (Royle ex D. Don) Royle. (Pinaceae) (SMH-401, AJK-233)	Baddul (KAS) Partal, Palundar (AJK)	Root Bark	Boiled in water and used as tea.	Y	N	N	Y	Gujjar, Pahari, Bakarwal	0.20	189
2	*Acacia modesta* Wall. (Fabaceae) (AJK-234)	Plai (AJK)	Gum	Chewed raw as gum.	N	N	N	Y	Gujjar, Pahari	0.22	215
3	*Aconitum heterophyllum* Wall. ex Royle (Ranunculaceae) (SMH-405)	Patrees, Atees (KAS)	Leaves	Cooked as vegetables.	Y	N	N	N	Gujjar, Pahar, Bakarwal	0.23	216
4	*Acorus calamus* L. (Asteraceae) (SMH-407)	Bariyan, Bareen (KAS) Shu-dag-nag-po (LAD)	Rhizome	Added in tea to enhance flavour.	Y	N	Y	N	Kashmiri, Gujjar, Pahari, Balti, Brokpa	0.18	175
5	*Aesculus indica* (Wall. ex Cambess.) Hook. (Sapindaceae) (SMH-110)	Goon, Haandoon, Khanor (KAS)	Fruit	Fruits are crushed and making flour.	Y	N	N	N	Gujjar, Pahari, Bakarwal	0.20	188
6	*Allium semenovii* Regel. (Amaryllidaceae) (SMH-413)	Wan pran, Ek pothi lahsun (KAS)	Leaves Root	Leaves are used as vegetables and roots	Y	N	N	N	Kashmiri, Gujjar,	0.25	239

				are used as spice and condiment. Muslim scholars probit the use before prayers.					Pahari, Bakarwal		
7	*Amaranthus viridis* L. (Amaranthaceae) (SMH- 303, AJK-235)	Kanhaar, Ganar, Ghanar (KAS, AJK, JAM)	Leaves	Boiled, fried and consumed as vegetable.	Y	Y	N	Y	Kashmiri, Gujjar, Pahari, Dogra	0.30	285
8	*Anethum graveolens* L. (Apiaceae) (AJK-236)	Soe, Sowa, Dill (AJK)	Leaves	Cooked as vegetable, and used as flavoring in salads.	N	N	N	Y	Kashmiri, Gujjar, Pahari	0.18	168
9	*Arnebia bethemii* (Wall. ex G. Don.) Johnst. (Boraginaceae) (SMH-304, AJK-237)	Ghaozban (KAS) Kahzawain (AJK, JAM)	Root	Used to make tea; same tea is also used to cure pneumonia and flu.	Y	Y	N	Y	Kashmiri, Gujjar, Pahari, Bakarwal, Dogra	0.31	299
10	*Arnebia euchroma* Royle. (Boraginaceae) (SMH-418, AJK-238)	Ratanjog (KAS, AJK, JAM) Demok, Dre mog, Bri-mog (LAD)	Roots Leaves	Roots are used as spices, and leaves are taken with tea to enhance flavor.	Y	N	Y	Y	Kashmiri, Gujjar, Pahari, Bakarwal, Brokpa, Beda	0.24	234
11	*Artemisia gmelinii* var. *gmelinii* Weber ex Steckm. (Asteraceae)	Bur tsemkhan pa (LAD)	Leaves Flowers	Leaves and flowers are mixed with wheat	N	N	Y	N	Brokpa, Beda, Balti	0.23	217

	(SMH-305)			flour and water to prepare ferments.							
12	*Asparagus gracilis* Royl. (Asparagaceae) (AJK-239)	Shagandal (AJK)	Whole plant	Used as vegetable.	N	N	N	Y	Gujjar, Pahari,	0.21	200
13	*Astragalus graveolens* Benth. (Fabaceae) (AJK-240)	Yurikund (AJK)	Fruit	Eaten as raw.	N	N	N	Y	Gujjar, Pahari	0.20	195
14	*Bauhinia variegata* L. (Fabaceae) (AJK-241)	Kalyari (JAM), Katchnar, Kachnar (AJK)	Flower	Cooked as vegetable.	N	Y	N	Y	Gujjar, Pahari, Bakarwal, Dogra	0.18	179
15	*Berberis asiatica* Roxb. ex DC. (Berberidaceae) (SMH-420)	Kaimbli (KAS), Kingora (JAM)	Fruit	Eaten raw.	Y	Y	N	N	Gujjar, Pahari, Bakarwal	0.30	285
16	*Berberis lycium* Royle (Berberidaceae) (SMH-306, AJK-242)	Kaimbli (KAS), Kingora (JAM), Sumbloo (AJK)	Fruit	Ripe bluish fruits are consumed raw.	Y	Y	N	Y	Gujjar, Pahari, Bakarwal	0.26	246
17	*Bergenia ciliata* (Haw.) Sternb. (Saxifragaceae) (SMH-202)	Palfut, Zakhmehayat (KAS), Pshanbheda(JAM)	Leaves Rhizome	Leaves are cooked as food. Rhizomes are used as herbal tea.	Y	Y	N	N	Kashmiri, Gujjar, Pahari, Bakarwal	0.27	256
18	*Bergenia stracheyi* (Hook. f. & Thomson) Engl. (Saxifragaceae)	Sapdotri (JAM), Katkotar (KAS)	Rhizome	Boiled to yield a decoction and taken as tea substitute.	Y	Y	N	N	Kashmiri, Gujjar,	0.25	243

	(SMH-423)								Pahari, Bakarwal		
19	*Betula utilis* D. Don. (Betulaceae) (SMH-425)	Bhojpatra, Burza (KAS)	Bark	Boiled in water for making herbal tea.	Y	N	N	N	Kashmiri, Gujjar, Pahari, Bakarwal	0.22	214
20	*Bombax ceiba* L. (Malvaceae) (SMH-307)	Simbal (JAM)	Flower	Cooked as vegetable and used for making tea.	N	Y	N	N	Gujjar, Pahari, Bakarwal, Dogra	0.17	165
21	*Brassica campestris* L. (Brassicaceae) (SMH-523, AJK-243)	Sarsoon (JAM), Tailgogal (KAS), Sarryan (AJK)	Leaves Seed	Leaves are cooked as vegetables. Oil is used in cooking.	Y	Y	N	Y	Kashmiri, Gujjar, Pahari	0.25	243
22	*Bunium persicum* Bioss. (Apiaceae) (SMH-308)	Kala zeera (JAM) Zuur (KAS)	Seed	Used as flavouring agent in different dishes.	Y	Y	N	N	Kashmiri, Gujjar, Pahari, Bakarwal, Dogra	0.33	315
23	*Capparis spinosa* L. (Capparaceae) (SMH-160)	Kabra (LAD)	Fruit	Unripe fruits are cooked as vegetables.	N	N	Y	N	Balti, Brokpa, Beda	0.22	213
24	*Capsella bursa-pastoris* (L.) Medik. (Brassicaceae) (SMH-427)	Kralmond (KAS), Hiri halian(JAM), Shamsho(LAD)	Leaves Shoots	Cooked as vegetable.	Y	Y	Y	N	Kashmiri, Gujjar, Pahari, Dogra,	0.37	354

									Balti, Brokpa, Beda		
25	*Caralluma edulis* (Edgew.) Bth. and Hk. (Asclepiadaceae) (AJK-244)	Chunga, Pippa (AJK)	Whole plant	Cooked as vegetable.	N	N	N	Y	Kashmiri, Gujjar Pahari	0.22	212
26	*Carissa spinarum* L (Apocynaceae) (AJK-245)	Ganrada (AJK)	Fruit	Eaten raw.	N	N	N	Y	Bakarwal, Gujjar Pahari	0.20	189
27	*Carissa carandas* L. (Apocynaceae) (SMH-310)	Karonda, Garna (JAM)	Fruit	Eaten raw.	N	Y	N	N	Gujjar, Pahari, Dogra	0.20	195
28	*Carum carvi* L. (Apiaceae) (SMH-104)	Kosnyot, Go-snyod (LAD)	Seed	Used as flavoring agent in a variety of dishes.	N	N	Y	N	Balti, Brokpa	0.19	185
29.	*Cassia fistula* L (Fabaceae) (SMH-311)	Korangal (JAM)	Flower	Cooked as vegetables.	N	Y	N	N	Bakarwal, Dogra	0.21	199
30	*Celtis australis* L. (Cannabaceae) (SMH-430)	Bramij, Bramiji (KAS)	Fruit	Eaten raw.	Y	N	N	N	Kashmiri, Pahari, Bakarwal	0.25	237
31	*Chenopodium album* L. (Chenopodiaceae) (SMH-430, AJK-246)	Tangthour chonma, Sneu (LAD), Bathwa (AJK), Bathu (KAS)	Leaves	Cooked as vegetable.	Y	N	Y	Y	Kashmiri, Gujjar, Pahari, Bakarwal,	0.32	310

									Balti, Brokpa		
32	*Chenopodium murale* L. (Chenopodiaceae) (AJK-247)	Bathwa (AJK)	Leaves	Cooked as vegetable.	N	N	N	Y	Kashmiri, Pahari, Bakarwal	0.33	315
33	*Cichorium intybus* L. (Asteraceae) (SMH-312, AJK-248)	Handh (KAS), Bathwa (AJK)	Leaves	Cooked as a vegetable and also used to make tea.	Y	N	N	Y	Kashmiri, Bakarwal, Gujjar, Pahari	0.37	350
34	*Cissus carnosa* (L) Lamk. (Vitaceae) (AJK-249)	Dakh (AJK)	Fruit	Eaten raw.	N	N	N	Y	Kashmiri, Gujjar, Pahari	0.25	241
35	*Citrus acida* L. (Rutaceae) (AJK-250)	Khatta (AJK)	Fruit	Used to make pickles.	N	N	N	Y	Gujjar, Pahari, Bakarwal,	0.22	211
36	*Commelina benghalensis* L. (Commelinaceae) (AJK-251)	Kanchara (AJK), Kanteri (JAM)	Leaves	Cooked as vegetable.	N	Y	N	Y	Gujjar, Dogra	0.22	208
37	*Conyza canadensis* (L.) Cronquist. (Asteraceae) (AJK-252)	Kutahudy (AJK)	Young leaves Seedlings	Young leaves and seedlings are cooked with rice.	N	N	N	Y	Kashmiri, Gujjar, Pahari	0.24	231
38	*Coriandrum sativum* L. (Apiaceae) (SMH-315, AJK-253)	Daanvaal (KAS), Dhanya (JAM, AJK)	Leaves Shoots	Used in curries to enhance flavor.	Y	Y	N	Y	Pahari, Gujjar, Bakarwal, Brokpa	0.33	316

39	*Dactylorhiza hatagirea* (D. Don) Soó. (Orchidaceae) (SMH-436)	Salam, Panja, Hathajari (KAS)	Tubers	The powder of dried tubers is mixed with ghee and eaten.	Y	N	N	N	Gujjar, Pahari, Bakarwal	0.22	214
40	*Delphinium brunonianum* Royle (Ranunculaceae) (SMH-105)	Byargodspos, Lunde aaown, Mask-larksupur (LAD)	Leaves Flowers	Leaves and flowers are mixed with wheat flour and water to prepare ferments.	N	N	Y	N	Brokpa, Balti	0.23	219
41	*Dioscorea deltoidea* Wall. ex Griseb. (Dioscoreaceae) (SMH-441)	Tard, Yam (KAS)	Leaves	Boiled and consumed as vegetable.	Y	N	N	N	Gujjar, Pahari, Bakarwal	0.23	220
42	*Dryopteris stewartii* Fraser-Jenk. (Dryopteridaceae) (SMH-443, AJK-254)	Kunji (AJK), Longad (KAS)	Leaves	Young leaves collected in spring are cooked as vegetable.	Y	N	N	Y	Kashmiri, Pahari,Gujjar, Bakarwal	0.30	285
43	*Duchesnea indica* (Jacks.) Focke. (Rosaceae) (SMH-316)	Sarpingdach (JAM) Zarkash (KAS)	Fruit	Fresh ripe fruits are eaten raw.	Y	Y	N	N	Kashmiri, Gujjar, Pahari, Bakarwal	0.23	218
44	*Elaeagnus angustifolia* L. (Elaeagnaceae) (AJK-255)	Konkol (AJK)	Fruit	Eaten raw.	N	N	N	Y	Kashmiri, Gujjar, Pahari	0.20	194
45	*Elaeagnus parvifolia* Wall. ex Royle (Elaeagnaceae) (AJK-256)	Kankoli (AJK)	Fruit	Used as a raw jam and preservative.	N	N	N	Y	Kashmiri, Gujjar, Pahari	0.18	168

46	*Elaeagnus umbellata* Thunb. (Elaeagnaceae) (SMH-317, AJK-257)	Kankoli (AJK, JAM)	Fruit	Eaten raw.	N	Y	N	Y	Gujjar, Pahari, Dogra	0.26	247
47	*Emblica officinalis* Gaertn. (Phyllanthaceae) (SMH-318)	Amla (JAM)	Fruit	Eaten raw.	N	Y	N	N	Kashmiri, Pahari, Gujjar, Dogra	0.20	194
48	*Eremurus himalaicus* Baker. (Asphodelaceae) (SMH-446)	Sheil-haakh (KAS), Vellun (KAS)	Leaves	Boiled, cooked like cabbage, and used as vegetable.	Y	N	N	N	Gujjar, Paharil, Bakarwal	0.23	217
49	*Fagopyrum esculentum* Moench. (Polygonaceae) (SMH-449)	Tromba (KAS, JAM), Dyat chonma (LAD)	Leaf	Boiled and used as vegetable.	Y	Y	Y	N	Kashmiri, Pahari, Gujjar, Brokpa, Balti	0.23	218
50	*Ficus carica* L. (Moraceae) (AJK-258)	Tosi, Tossa (AJK)	Fruit	Eaten fresh and sometimes dried for later use.	N	N	N	Y	Pahari, Gujjar,	0.30	285
51	*Ficus palmata* Roxb. (Moraceae) (SMH-319, AJK-259)	Anjeer (JAM), Kemri (AJK)	Fruit	Eaten fresh and sometimes dried for later use.	N	Y	N	Y	Gujjar, Pahari, Dogra	0.23	216
52	*Flacourtia indica* (Burm.) Merrill (Flacourtiaceae) (SMH-320, AJK-260)	Kanju (JAM), Kakoh (AJK)	Fruit	Eaten raw.	N	Y	N	Y	Gujjar, Pahari, Dogra	0.23	220

53	*Foeniculum vulgare* (Mill) (Apiaceae) (SMH-322)	Badyaan (KAS), Saunf (JAM)	Seed	Used as flavoring agent in different dishes.	Y	Y	N	N	Kashmiri, Gujjar Pahari, Dogra	0.33	318
54	*Fragaria nubicola* (Lindl. ex Hook. f.) Lacaita (Rosaceae) (SMH-450, AJK-261)	Jangli-gonch (KAS), Buddha Mewa (JAM, AJK)	Fruit	Eaten with craze because of pleasant strawberry flavor. And roots are used to make herbal tea local Pahari, Bakarwal people	Y	Y	N	Y	Kashmiri, Pahari, Gujjar, Bakarwal	0.34	324
55	*Galinsoga parviflora* Cav. (Asteraceae) (SMH-323)	Mirchghas (KAS), Galinsoga (JAM)	Leaves	Cooked and fried with spices as vegetable.	Y	Y	N	N	Kashmiri, Pahari Gujjar, Bakarwal	0.22	216
56	*Heracleum candicans* Wall.ex DC. Apiaceae (SMH-454)	Mirkul (KAS)	Leaves	Used as salad especially by Bakarwal people.	Y	N	N	N	Gujjar, Pahari, Bakarwal	0.25	241
57	*Hippophae rhamnoides* L. (Eleagnaceae) (SMH-156)	Sastalulu, Tshogskyur, Star bu (LAD)	Fruit	Eaten raw and nowadays a juice is industrially prepared from them.	N	N	Y	N	Beda, Balti, Brokpa	0.22	218
58	*Juglans regia* L (Juglandaceae) (SMH-326, AJK-262)	Dun (KAS), Akhrot, Khoad (AJK)	Kernel Walnut	Kernels are eaten raw, and oil is used in cooking. male inflorescence is boiled	Y	N	N	Y	Kashmiri, Gujjar, Pahari, Bakarwal	0.33	316

			male Inflorescence	dried and used as vegetables in winter season							
59	*Lamium amplexicaule* L. (Lamiaceae) (SMH-327, AJK-263)	Godrii (AJK, JAM)	Leaves	Used as vegetable.	N	Y	N	Y	Gujjar, Pahari, Dogri	0.23	220
60	*Leucas cephalotes* (Roth.) Spreng. (Lamiaceae) (AJK-264)	Chara, Gut dode, Guma (AJK)	Leaves Shoot	Cooked as vegetable.	N	N	N	Y	Gujjar, Pahari	0.22	216
61	*Luffa cylindrica* (L.) M. Roem. (Cucurbitaceae) (SMH-328, AJK-265)	Toori (AJK, JAM)	Fruit	Cooked as vegetable.	N	Y	N	Y	Gujjar, Pahari, Dogri	0.22	213
62	*Malva neglecta* Wallr. (Malvaceae) (SMH-465)	Sonchal (KAS), Suchal Sabzi (JAM)	Leaves	Cooked and used as vegetable, also used as salad.	Y	Y	N	N	Kashmiri, Pahari, Gujjar, Bakarwal, Dogri	0.33	316
63	*Malva parviflora* L. (Malvaceae) (AJK-267)	Sonchal (AJK)	Leaves	Cooked as vegetable.	N	N	N	Y	Gujjar, Pahari	0.33	318
64	*Malva verticillata* L. (Malvaceae) (SMH-329)	Icham-pa (LAD)	Leaves	Cooked as vegetable.	N	N	Y	N	Brokpa, Balti	0.29	279
65	*Medicago polymorpha* L. (Fabaceae) (AJK-268)	Sriri, Sri (AJK)	Leaves	Cooked as vegetable.	N	N	N	Y	Gujjar,Pahari	0.22	210

66	*Mentha arvensis* L. (Lamiaceae) (SMH-467)	Pudun (KAS), Podina (JAM)	Leaves	Used as spice and condiments, also used to make paste called chutney"	Y	Y	N	N	Kashmiri, Pahari, Dogra	0.34	328
67	*Mentha longifolia* L. (Lamiaceae) (SMH-330, AJK-269)	Pudun, ChittaPotna (KAS, AJK), Podina (JAM)	Leaves	Used as spice and condiments, also used to make paste called chutney	Y	Y	N	Y	Kashmiri, Pahari, Dogra	0.26	246
68	*Micromeria biflora* (Ham.) Bth. (Lamiaceae) (AJK-270)	Narayshamakay, Ban ajwain (AJK)	Flower Leaves	Dried flowers and young leaves are used to make tea and flavoring agent in curries.	N	N	N	Y	Kashmiri, Pahari	0.20	196
69	*Momordica dioica* Roxb. ex Willd. (Cucurbitaceae) (SMH-331)	Kokora (JAM)	Fruit	Cooked as vegetable.	N	Y	N	N	Gujjar,Pahari,Dogra,	0.21	199
70	*Morus alba* L. (Moraceae) (SMH-334)	Tul, Toot (KAS), Dar shing (LAD)	Fruit	Eaten raw.	Y	N	Y	N	Kashmiri, Gujjar, Pahari, Balti, Brokpa	0.29	279
71	*Morus nigra* L. (Moraceae) (SMH-335)	Tul, Shah toot (KAS), Sang (LAD)	Fruit	Dark red fruits are eaten raw.	Y	N	Y	N	Kashmiri, Gujjar Pahari, Balti, Brokpa	0.31	295

72	*Nasturtium officinale* R. Br (Brassicaceae) (AJK-271)	Taramira (AJK)	Leaves	Cooked as vegetable.	N	N	N	Y	Gujjar, Bakarwal	0.28	265
73	*Nepeta floccosa* Benth. (Lamiaceae) (SMH-153)	Shamalolo, Shngukram (LAD)	Leaves Stem	The dried leaves and shoots are used to flavor local dishes.	N	N	Y	N	Brokpa, Balti	0.29	275
74	*Olea ferruginea* Royle (Oleaceae) (AJK-272)	Kahu (AJK)	Leaves	Used to make tea	N	N	N	Y	Kashmiri, Gujjar Pahari	0.22	210
75	*Ocimum basilicum* L. (Lamiaceae) (SMH-336)	Baboori, Babar (KAS), Maling (JAM)	Seed	Used in making beverages called "sharbat" to beat summer heat.	Y	Y	N	N	Kashmiri, Gujjar, Pahari, Dogri	0.21	200
76	*Onopordum acanthium* L (Asteraceae) (AJK-273)	Kandyara (AJK)	Whole plant	Leaves and young plants are cooked as vegetables.	N	N	N	Y	Gujjar, Pahari	0.25	235
77	*Oenothera rosea* L. (Onagraceae) (AJK-274)	Jungli gulab (AJK)	Root Shoot	Young roots are cooked as vegetable (with a peppery flavour). Shoots are used as a salad.	N	N	N	Y	Gujjar, Pahari	0.21	199
78	*Origanum vulgare* L. (Lamiaceae) (SMH-522, AJK-275)	Ganeyar (KAS), Banjawain (AJK)	Leaves	Cooked as vegetable, often used as a salad, dried leaves are boiled in water to make herbal tea.	Y	N	N	Y	Kashmiri, Gujjar, Pahari	0.25	237

79	*Oxalis corniculata* L. (Oxalidaceae) (AJK-276)	Khatibuti (AJK)	Leaves	Cooked as vegetable.	N	N	N	Y	Gujjar, Pahari	0.24	234
80	*Oxyria digyna* Hill (Polygonaceae) (SMH-337)	Lamanchu, Chu lcum (LAD)	Leaves	Cooked as vegetable.	N	N	Y	N	Balti, Brokpa	0.21	198
81	*Phylanthus emblica* L. (Phyllanthaceae) (SMH-338, AJK-277)	Amla (JAM, AJK)	Fruit	Used to make pickles and sometimes eaten raw.	N	Y	N	Y	Gujjar, Pahari, Dogra	0.18	168
82	*Phytolacca acinosa* Roxb. (Phytolaccaceae) (SMH-473)	Hapat-makai (KAS)	Leaves	Cooked and used as vegetable.	Y	N	N	N	Gujjar, Pahari, Bakarwal	0.24	226
83	*Plantago depressa* Willd. (Plantaginaceae) (SMH-140)	Tha-ram (LAD)	Leaves	Boiled and used as vegetable.	N	N	Y	N	Brokpa, Balti, Beda	0.22	210
84	*Plantago lanceolata* L. (Plantaginaceae) (SMH-478)	Gul (KAS)	Leaves	Boiled and used as vegetable.	Y	N	N	N	Kashmiri	0.33	319
85	*Plantago major* L. (Plantaginaceae) (SMH-479)	Bud-gull (KAS)	Leaves	Cooked as vegetable especially by Gujjar people.	Y	N	N	N	Kashmiri, Gujjar, Pahari	0.34	327
86	*Podophyllum hexandrum* Royle (Berberidiaceae) (SMH-481)	Wanwagun, Van Kakdi (KAS), Hol-mo-se,	Fruit	Eaten as raw especially by Gujjar, Pahari people.	Y	N	Y	N	Kashmiri, Gujjar, Pahari,Bed a	0.36	345

		Denmokushu (LAD)									
87	*Polygonum aviculare* L. (Polygonaceae) (SMH-151)	Endrani (LAD) Drub (KAS)Maachran chai (JAM)	Leaves	Cooked as vegetable especially by Gujjar, Bakarwal people.	Y	Y	Y	N	Kashmiri, Gujjar, Pahari, Bakarwal Brokpa, Balti	0.23	219
88	*Polygonum nepalense* Meissn. (Polygonaceae) (AJK-278)	Hulla (AJK)	Leaves Shoot	Young leaves, shoots eaten raw, cooked as vegetable.	N	N	N	Y	Gujjar, Pahari	0.23	218
89	*Polygonum amplexicaule* D. Don (Polygonaceae) (SMH-482, AJK-279)	Adder (JAM, AJK), Maachran (KAS)	Rhizomes Stem	Used for making tea and vegetable.	Y	Y	N	Y	Gujjar, Pahari, Bakarwal	0.33	318
90	*Portulaca oleracea* L. (Portulacaceae) (SMH-339)	Nunar (KAS), Kulfa (JAM)	Leaves	Cooked as vegetable especially by Kashmiri, Pahari people.	Y	Y	N	N	Kashmiri, Gujjar, Pahari, Bakarwal, Dogra	0.36	346
91	*Prunus armeniaca* L., (Rosaceae) (SMH-340)	Khubani (KAS, JAM), Chuli, Chair (LAD)	Fruit	Eaten raw. Dry Chuli/Chair are boiled in water and used as vegetable especially by Balti people.	Y	Y	Y	N	Kashmiri, Gujjar, Pahari, Beda, Balti Brokpa	0.26	245

92	*Prunus cornuta* (Wall. ex Royle) Steud. (Rosaceae) (SMH-485)	Chuli (KAS)	Fruit	Eaten raw.	Y	N	N	N	Gujjar, Pahari	0.29	278
93	*Prunus domestica* L. (Rosaceae) (SMH-341)	Aloobukhra (JAM), Aar (KAS)	Fruit	Eaten raw.	Y	Y	N	N	Kashmiri, Gujjar Pahari, Dogra	0.30	282
94	*Punica granatum* L. (Lythraceae) (SMH-342, AJK-280)	Se-bru (LAD), Daroona (JAM), Aaanar (AJK), Daan (KAS)	Fruit	Eaten raw. In "Kashmiri" dried fruits are used as condiment by local chefs (Waza).	Y	Y	Y	Y	Kashmiri, Gujjar, Pahari, Bakarwal, Beda, Balti, Brokpa	0.33	312
95	*Pyrus pashia* Buch. Ham. ex D. Don (Rosaceae) (SMH-486)	Kainthi (JAM)	Fruit	Eaten raw.	N	Y	N	N	Gujjar, Pahari Bakarwal	0.29	274
96	*Pyrus pyrifolia* (Burm. f.) Nakai. (Rosaceae) (SMH-343)	Dandalli (JAM)	Fruit	Eaten raw.	N	Y	N	N	Gujjar,Pah ari	0.23	216
97	*Ranunculus sceleratus* L. (Ranunculaceae) (AJK-281)	Korekandoli (AJK) Chambelbooti (AJK)	Fruit	Eaten raw.	N	N	N	Y	Kashmiri, Gujjar, Pahari	0.20	188
98	*Rheum australe* D. Don (Polygonaceae) (SMH-345)	Pambhak (JAM),	Leaves	Cooked as vegetable.	Y	Y	N	N	Kashmiri, Gujjar,	0.36	342

		Pambchalan (KAS)							Pahari, Bakarwal		
99	*Rheum spiciforme* Royle (Polygonaceae) (SMH-167)	Lachhu (LAD) Chu rtsa (LAD)	Stem	Stem petioles and young stems are cooked as vegetables. Stem petioles is also chewed as raw by local people.	N	N	Y	N	Brokpa, Balti, Beda	0.23	218
100	*Rheum webbianum* Royle (Polygonaceae) (SMH-488)	Pambhak (KAS), Chutyal (JAM)	Leaves Stem	Cooked as vegetable. Raw stems are chewed by local Gujjar, Pahari, Bakarwal people	Y	Y	N	N	Kashmiri, Gujjar, Pahari, Bakarwal	0.25	238
101	*Rhodiola sacra (*Prain ex Raym. Hamet) S.H. Fu (Crassulaceae) (SMH-193)	Srolo-marpo (LAD)	Leaves	Cooked as vegetables.	N	N	Y	N	Brokpa, Balti, Beda	0.20	196
102	*Rhodiola imbricate* Edgew (Crassulaceae) (SMH-143)	Shro-Lo, Shrolo, ho-ngo-ngo (LAD)	Leaves	Used to make a unique dish called "Tantura".	N	N	Y	N	Brokpa, Balti, Beda	0.22	210
103	*Rhododendron arboreum* Sm. (Ericaceae) (SMH-347)	Gurans, Burans (JAM)	Flower	Cooked as vegetable and used in tea. Flowers are also used to please Lord Shiva.	N	Y	N	N	Gujjar, Pahari , akarwal, Dogra	0.23	218

104	*Rosa moschata* Herrm. (Rosaceae) (SMH-348)	Phulwadhi (JAM), Gulabposh (KAS)	Fruit	Eaten raw.	Y	Y	N	N	Kashmiri, Gujjar, Pahari, Dogra	0.30	285
105	*Rosa sericea* Lindl. (Rosaceae) (SMH-149)	Se ba (LAD)	Fruit	Eaten raw.	N	N	Y	N	Balti, Beda, Brokpa	0.26	246
106	*Rosa webbiana* Wallich ex Royle (Rosaceae) (SMH-204)	Siah, Se ba (LAD), Gulab (KAS)	Fruit	Eaten by children, also used to prepare jam.	Y	N	Y	N	Kashmiri, Gujjar Pahari, Balti,Beda	0.29	275
107	*Rubus ellipticus* Sm. (Rosaceae) (SMH-349, AJK-282)	Akhray (KAS), Peelaakra (JAM, AJK)	Fruit	Eaten raw.	Y	Y	N	Y	Gujjar, Pahari	0.21	198
108	*Rubus fruticosus* Wallich. (Rosaceae) (AJK-283)	Akhari (AJK)	Fruit	Eaten raw.	N	N	N	Y	Kashmiri, Gujjar Pahari	0.26	246
109	*Rubus niveus* Thunb. (Rosaceae) (AJK-284)	Pagnar (AJK)	Fruit	Eaten raw.	N	N	N	Y	Gujjar, Pahari	0.29	275
110	*Rubus ulmifolius* Schott (Rosaceae) (SMH-350)	Akhray (KAS, JAM)	Fruit	Eaten raw.	Y	Y	N	N	Gujjar, Pahari, Bakarwal Dogra	0.26	249
111	*Rumex dentatus* L. (Polygonaceae)	Abij (KAS), Abugee (JAM)	Leaves	Cooked as vegetable.	Y	Y	N	N	Kashmiri, Gujjar,	0.21	197

	(SMH-491)								Pahari, Bakarwal		
112	*Rumex hastatus* Baldwin (Polygonaceae) (SMH-351)	Aula (KAS), Khatimal (JAM)	Leaves	Eaten as salad. Sometime leaves are grinded mixed with salt to form paste like substance called "chuteny" which s then taken with other foods.	Y	Y	N	N	Kashmiri, Gujjar Pahari,Bak arwal Dogra	0.23	218
113	*Rumex nepalensis* Spreng. (Polygonaceae) (SMH-492, AJK-285)	Jungli palak (KAS), Ulla (JAM)Sho-mang (LAD), Holla (AJK)	Leaves	Cooked as vegetable.	Y	Y	Y	Y	Gujjar, Pahari, Bakarwal, Dogra, Brokpa	0.34	321
114	*Sambucus wightiana* Wall. ex Wight & Arn. (Adoxaceae) (SMH-495, AJK-286)	Gandi (KAS), Phakloon (AJK)	Fruit	Eaten raw by local Gujjar, Pahari people.	Y	N	N	Y	Gujjar, Pahari, Bakarwal	0.26	247
115	*Saussurea costus* (Falc.) Lipsch. (Asteraceae) (SMH-352, AJK-287)	Kuth (KAS, AJK)	Leaves	Cooked with spices as vegetable.	Y	N	N	Y	Gujjar, Pahari, Bakarwal	0.31	295
116	*Sesamum orientale* L. (Pedaliaceae) (SMH-353)	Til (JAM)	Seed	Eaten raw and applied on the bread to enhance taste.	N	Y	N	N	Gujjar, Pahari, Bakarwa, lDogra	0.20	188

117	*Solanum nigrum* L. (Solanaceae) (SMH-354, AJK-288)	Makoi (KAS), Kachmach (JAM), Maki (AJK)	Fruit	Cooked as vegetable.	Y	Y	N	Y	Gujjar, Pahari Bakarwal	0.18	176
118	*Sonchus asper* (L.) Hill. (Asteraceae) (AJK-289)	Dodak (AJK)	Whole plant	Young leaves and stem are cooked as vegetable.	N	N	N	Y	Gujjar, Pahari, Bakarwal	0.27	256
119	*Stellaria media* (L.) Vill. (Caryophyllaceae) (SMH-355)	Nick hakh (KAS), Koku (JAM)	Leaves	Cooked as vegetable at tender stage.	Y	Y	N	N	Kashmiri, Gujjar, Pahari, Dogra	0.33	314
120	*Taraxacum officinale* (L.) Weber ex F.H. Wigg. (Asteraceae) (SMH-504, AJK-290)	Hand (KAS, AJK), Han, Khorma (LAD), Handri (JAM)	Leaves	Cooked as vegetable; herbal tea is prepared from fresh leaves.	Y	Y	Y	Y	Kashmiri, Gujjar, Pahari, Bakarwal, Dogra, Balti, Brokpa	0.36	346
121	*Taxus wallichiana* Zucc. (Taxaceae) (SMH-505)	Bririmi, Oostul (KAS)	Bark	Boiled in water and taken as tea.	Y	N	N	N	Gujjar, Pahari, Bakarwal	0.25	241
122	*Terminalia bellirica* (Gaertn.) Roxb. (Combretaceae) (SMH-356)	Bhera, Bedda (JAM)	Fruit	Pulp of fruit is eaten raw.	N	Y	N	N	Pahari, Dogra	0.23	219

123	*Thymus linearis* Benth. (Lamiaceae) (SMH-506)	Baljaween (JAM), Tumbrak (KAS)	Whole plant	After softening whole plant by pounding on a stone slab is used in making "achar" (Pickle).	Y	Y	N	N	Gujjar, Pahari, Bakarwal	0.30	285
124	*Trichosanthes cucumerina* L. (Cucurbitaceae) (SMH-357)	Parhul (JAM)	Fruit	Cooked as vegetable.	N	Y	N	N	Gujjar, Pahari, Bakarwal	0.26	251
125	*Trifolium pratense* L. (Fabaceae) (SMH-507)	Pawera (KAS, JAM)	Leaves	Cooked and used as vegetable. Rarely used as salad.	Y	Y	N	N	Gujjar, Pahari, Bakarwal	0.23	216
126	*Trifolium repens* L. (Fabaceae) (SMH-508)	Chopati (KAS, JAM)	Leaves	Eaten as salad, and cooked as vegetable.	Y	Y	N	N	Gujjar, Pahari, Bakarwal	0.23	218
127	*Trillium govanianum* Wall. ex D. Don (Melanthiaceae) (SMH-509)	Tripater, Nagchhatri, Satva (KAS)	Leaves	Boiled and used as vegetable.	Y	N	N	N	Kashmiri, Gujjar, Pahari, Bakarwal	0.29	274
128	*Tussilago farfara* L. (Asteraceae) (AJK-291)	Bann Hulla (AJK)	Flower	Flower buds are cooked as vegetable.	N	N	N	Y	Gujjar, Pahari, Bakarwal	0.25	235
129	*Urtica dioica* L. (Urticaceae) (SMH-510)	Soi (KAS), Kayyari (JAM)	Leaves	Cooked as vegetable. Tea is also prepared from fresh leaves.	Y	Y	N	N	Kashmiri, Gujjar, Pahari, Bakarwal	0.31	298

130	*Urtica hyperborea* Jacquem. ex Wedd (Urticaceae) (SMH-171)	Dzatsutt, Zwa (LAD)	Leaves	Used to prepare soup.	N	N	Y	N	Beda, Brokpa, Balti	0.22	214
131	*Verbascum thapsus* L. (Scrophulariaceae) (SMH-511)	Gamhar, Mullei (KAS)	Seed	Eaten with ghee.	Y	N	N	N	Gujjar, Pahari	0.29	274
132	*Viburnum grandiflorum* Wall. ex DC. (Adoxaceae) (SMH-358, AJK-292)	Jammar, Kulmach (JAM), Guch (AJK)	Fruit	Eaten raw.	Y	Y	N	Y	Kashmiri, Gujjar, Pahari, Bakarwal, Dogra	0.34	326
133	*Viola odorata* L. (Violaceae) (SMH-512)	Nun posh (KAS), Karpo (JAM)	Leaves	Boiled then fried with spices and taken as vegetable.	Y	Y	N	N	Kashmiri, Gujjar Pahari, Bakarwal	0.22	214
134	*Vitis jacquemontii* R. Parker. (Vitaceae) (SMH-359, AJK-293)	Daakh (JAM, AJK), Gandum (LAD), Jungli Dach, Dach (KAS)	Fruit Leaves	Eaten raw and young leaves are cooked as vegetable.	Y	Y	Y	Y	Kashmiri, Gujjar, Pahari, Balti, Beda, Brokpa	0.23	218
135	*Zanthoxylum armatum* DC. (Rutaceae) (SMH-515)	Timbru, Timbur (JAM)	Fruit	Grinded with some other herbs like mint to make a paste like substance called “chutney” which is	N	Y	N	N	Gujjar, Pahari, Dogra	0.25	241

				used to enhance taste of meals.							
136	*Zanthoxylum aromatus* DC (Rutaceae) (AJK-294)	Timbru (AJK), Timbe (AJK)	Fruit	Grinded with some other herbs like mint to make a paste like substance called "chutney" which is used to enhance taste of meals.	N	N	N	Y	Gujjar, Pahari, Bakarwal	0.23	222
137	*Ziziphus jujuba* Lamk (Rhamnaceae) (SMH-360, AJK-295)	Beree (AJK), Jand-beri (JAM)	Fruit	Eaten raw.	N	Y	N	Y	Gujjar, Pahari, Bakarwal, Dogra	0.20	195
138	*Ziziphus nummularia* (Burm. f.) Wight &Arn. (Rhamnaceae) (AJK-296)	Bair (AJK)	Fruit	Eaten raw.	N	N	N	Y	Gujjar, Pahari, Bakarwal	0.25	236
139	*Ziziphus oxyphylla* Edgew. (Rhamnaceae) (SMH-361)	Kokanberii (JAM)	Fruit	Eaten raw.	N	Y	N	N	Gujjar, Pahari, Bakarwal, Dogra	0.24	229
Fungi											
140	*Rhizopogon luteolus* Fr. & Nordholm (Rhizopogonaceae) (SMH-346)	Dudhkutt, Kundi, Kutch, Gav pagur (KAS)	Fruiting body	Consumed as food (Cooked as well as raw).	Y	N	N	N	Kashmiri, Gujjar, Pahari	0.24	228

141	*Ramaria formosa* (Pers.) Quél. (Gomphaceae) (SMH-344)	Panzanguj, Hapatpaan (KAS)	Fruiting body	Cooked as vegetable.	Y	N	N	N	Kashmiri, Gujjar Pahari	0.28	265
142	*Morchella esculenta* L. (Morchellaceae) (SMH-332)	Guchi (JAM), Guch (KAS)	Fruiting body	Cooked, roasted, and used as vegetable.	Y	Y	N	N	Kashmiri, Gujjar, Pahari, Dogra	0.24	234
143	*Morchella vulgaris* (Pers.) Boud. (Morchellaceae) (SMH-333)	Kannguch, Guuch (KAS)	Fruiting body	Cooked and used as vegetable.	Y	N	N	N	Kashmiri, Gujjar, Pahari	0.28	265
144	*Geopora arenicola* Lev. Kers (Pyronemataceae) (SMH-453)	Shazikan (KAS)	Fruiting body	Cooked as vegetable.	Y	N	N	N	Kashmiri, Pahari, Gujjar, Bakarwal	0.33	318
145	*Gyromitra esculenta* (Pers.) Fr. (Discinaceae) (SMH-324)	Kankuch (KAS)	Fruiting body	Cooked as vegetable.	Y	N	N	N	Kashmiri, Pahari, Gujjar, Bakarwal	0.22	215
146	*Gyromitra sphaerospora* (Peck) Sacc. (Discinaceae) (SMH-325)	Kankuch, Kanpapdi (KAS)	Fruiting body	Cooked as vegetable.	Y	N	N	N	Kashmiri, Pahari, Gujjar, Bakarwal	0.21	200
147	*Flammulina velutipes* (Curt.) Singer (Mazasmiaceae) (SMH-321)	Hend, Drubdi (KAS)	Fruiting body	Consumed as vegetable especially by Pahari people.	Y	N	N	N	Kashmiri, Pahari, Bakarwal	0.20	195

148	*Coprinus atramentarius* (Bull. Fr.) Fr. (Coprinaceae) (SMH-313)	Sakerbub, Sakerboob (KAS)	Fruiting body	Cooked as vegetable.	Y	N	N	N	Kashmiri, Pahari Gujjar, Bakarwal	0.20	198
149	*Coprinus comatus* (O.F. Müll.) Pers. (Coprinaceae) (SMH-314)	Setherwat (KAS)	Fruiting body	Cooked as vegetable during the early stages.	Y	N	N	N	Pahari, Gujjar, Bakarwal	0.22	210
150	*Calocera viscose* (Pers.) Fr. (Dacrymycetaceae) (SMH-309)	Paunzaunguje (KAS)	Fruiting body	Cooked as vegetable	Y	N	N	N	Kashmiri, Gujjar, Pahari, Bakarwal	0.23	224
151	*Agaricus bisporus* (Lange) Bach. (Agaricaceae) (SMH- 301)	Maaz hadur, Haind (KAS)	Fruiting body	Cooked as vegetable.	Y	N	N	N	Kashmiri, Gujjar, Pahari, Bakarwal	0.27	256
152	*Agaricus campestris* (L.) Fr. (Agaricaceae) (SMH- 302)	Maazhhadur (KAS)	Fruiting body	Cooked as vegetable.	Y	N	N	N	Kashmiri, Gujjar, Pahari, Bakarwal	0.25	241
Mammals											
153	*Boselaphus tragocamelus* Blainville, 1816 (Bovidae)	Neelee gaan, Nail Gai (JAM)	Bush meat Trotters Heart Lungs Kidney	Meat is smoke-roasted, dried, and cooked, Trotters, heart, lungs, kidney, spleen are simply	N	Y	N	N	Gujjar, Pahari, Bakarwal	0.26	246

			Spleen	cooked and taken with rice.							
154	*Capra falconeri* Wagner, 1839 (Bovidae)	Markhor (JAM, KAS, AJK)	Bush meat Brain Trotters Heart Lungs Kidney Spleen	Meat is smoke-roasted, dried, and cooked. Brain is fried. Trotters, heart, lungs, kidney, spleen are cooked and eaten with rice and Naan (bread).	Y	Y	N	Y	Gujjar, Pahari, Bakarwal	0.32	310
155	*Capra sibirica* Pallas, 1776 (Bovidae)	Jangli bakri (AJK), Killbakri (JAM), Skin (LAD)	Bush meat Brain Trotters Heart Lungs Kidney Spleen	Meat is smoke-roasted dried, and cooked. Brain is fried. Trotters, heart, lungs, kidney, spleen are cooked and eaten with rice and Naan.	N	Y	Y	Y	Gujjar, Pahari, Bakarwal, Beda, Balti, Brokpa	0.29	274
156	*Cervus elaphus hanglu* Wagner, 1844 (Cervidae)	Hangul (KAS)	Bush meat Trotter Heart Lungs Kidney Spleen	Meat is smoke-roasted dried, and cooked. Trotters, heart, lungs, kidney, spleen are cooked and eaten with rice and Naan.	Y	N	N	N	Kashmiri	0.32	310

157	*Hemitragus jemlahicus* Hodgson, 1841 (Bovidae)	Karth, Jagla (JAM)	Bush meat Trotters Heart Lungs Kidney Spleen	Meat is smoke-roasted, dried, and cooked. Trotters, heart, lungs, kidney, spleen are cooked, fried and eaten.	N	Y	N	N	Gujjar, Pahari, Bakarwal	0.24	225
158	*Lepus oiostolus* Hodgson, 1840 (Leporidae)	Ribong (LAD), Jangli-khargoosh (JAM)	Bush meat	Cooked, fried and eaten.	N	Y	Y	N	Gujjar, Pahari, Bakarwal, Beda, Balti	0.25	241
159	*Marmota himalayana* Hodgson, 1841 (Sciuridae)	Phia (LAD)	Bush meat	Cooked, fried and eaten.	N	N	Y	N	Balti, Beda, Brokpa	0.22	214
160	*Moschus moschiferus* L. 1758 (Cervidae)	Hern (KAS), Kusturee (JAM) (AJK)	Bush meat Trotters Heart Lungs Kidney	Meat is smoke-roasted dried, and cooked. Trotters, heart, lungs, kidney are cooked and eaten with Naan (bread) and riceor fried and eaten.	Y	Y	N	Y	Kashmiri, Gujjar, Pahari, Bakarwal	0.21	200
161	*Muntiacus muntjak* Zimmermann, 1780 (Cervidae)	Kakkar (KAS, JAM, AJK)	Bush meat	Smoke- roasted, dried, and cooked and eaten.	Y	Y	N	Y	Kashmiri, Gujjar, Pahari, Bakarwal	0.23	216

162	*Naemorhedus goral* Hardwicke, 1825 (Bovidae)	Pijjar (JAM) (AJK) Goral (AJK)	Bush meat Trotters Heart Lungs Kidney	Meat is smoke-roasted, dried, and cooked. Trotters, heart, lungs, kidney are cooked and eaten with Naan (bread), riceor fried and eaten.	N	Y	N	Y	Gujjar, Pahari Bakarwal	0.25	243
163	*Ovis ammon* L., 1758 (Bovidae)	Nyan (LAD)	Bush meat Trotters Heart Lungs Kidney Spleen	Meat is dried and cooked. Trotters, heart, lungs, kidney, spleen are cooked and eaten.	N	N	Y	N	Beda, Balti, Brokpa	0.22	210
164	*Ovis aries vignei* Blyth, 1841 (Bovidae)	Shapo (LAD)	Bush meat Trotters Heart Lungs Kidney Spleen	Meat is dried and cooked. Trotters, heart, lungs, kidney, spleen are cooked.	N	N	Y	N	Beda, Balti, Brokpa	0.29	275
165	*Procapra picticaudata* Hodgson, 1846 (Bovidae)	Goa (LAD)	Bush meat Trotters Heart	Meat is dried and cooked.	N	N	Y	N	Beda, Balti, Brokpa	0.23	220

			Lungs Kidney Spleen	Trotters, heart, lungs, kidney, spleen are cooked and eaten.							
166	*Pseudois nayaur* Hodgson, 1833 (Bovidae)	Napo (LAD)	Bush meat Trotters Heart Lungs Kidney Spleen	Meat is dried and cooked. Trotters, heart, lungs, kidney, spleen are cooked and eaten as such.	N	N	Y	N	Beda, Balti, Brokpa	0.32	305
Birds											
167	*Alectoris chukar* J.E. Gray, 1830 (Phasianidae)	Vankukur (KAS) Chukar (KAS) (JAM) Srakpa (LAD)	Bush meat Egg	Meat is cooked and eaten with rice or Naan (bread). Soup is also made from the fresh meat. Eggs are boiled or fried taken with tea.	Y	Y	Y	N	Kashmiri, Gujjar Pahari, Bakarwal Balti	0.37	356
168	*Anas acuta* L., 1758 (Anatidae)	Pechin (KAS)	Bush meat Gizzard	Meat is cooked and eaten with rice or Naan (bread). Soup is also made from the fresh meat. Gizzard is cooked and eaten.	Y	N	N	N	Kashmiri	0.32	306

				Eggs are boiled or fried and consumed with tea.							
169	*Columba livia,* Gmelin, 1789 (Columbidae)	Kotur (KAS) Kubutar (AJK) Mukron (LAD)	Bush meat Gizzard	Meat is cooked and eaten with rice or Naan (bread). Soup is also made from the gizzard.	Y	N	Y	Y	Kashmiri, Gujjar, Pahari, Balti, Beda, Braokpa	0.26	245
170	*Columba rupestris* Pallas, 1811 (Columbidae)	Mukron (LAD)	Bush meat Gizzard	Both meat and gizzard are cooked and eaten with rice or Naan (bread)	N	N	Y	N	Beda, Balti, Brokpa	0.22	213
171	*Hirundo rustica* L., 1758 (Hirundinidae)	Katij (KAS) Ababeel (AJK)	Bush meat	Cooked or fried and eaten with Naan (bread). In Azad Kashmir, the use of *Hirundo rustica* is prohibited by the religious scholars, saying that the species have protected the holy Kaba in past.	Y	N	N	Y	Kashmiri, Gujjar, Pahari	0.20	195
172	*Ixobrychus minutus* L., 1766 (Ardeidae)	Gui (KAS)	Bush meat	Cooked or fried and eaten with Naan (bread).	Y	N	N	N	Kashmiri	0.20	199

173	*Jynx torquilla* L., 1758 (Picidae)	Viri Mot (KAS)	Bush meat	Cooked or fried and eaten with Naan (bread).	Y	N	N	N	Kashmiri	0.22	210
174	*Lophophorus impejanus* Latham, 1790 (Phasianidae)	Wan Kokur, Jungli murga, Son Murg (KAS)	Bush meat Gizzard	Both meat and gizzard are cooked. Soup is made from the meat.	Y	N	N	N	Kashmiri, Gujjar, Pahari, Bakarwal	0.21	205
175	*Oenanthe oenanthe* L., 1758 (Sylviidae)	Tirki (AJK)	Bush meat	Cooked fried and eaten with Naan (bread).	N	N	N	Y	Kashmiri, Gujjar, Pahari	0.22	209
176	*Passer domesticus* L., 1758 (Passeridae)	Chaer (KAS) Chidrea (AJK)	Bush meat	Cooked fried and eaten with Naan (bread).	Y	N	N	Y	Kashmiri, Gujjar, Pahari	0.23	221
177	*Pavo cristatus* L., 1758 (Phasianidae)	Moor (JAM)	Bush meat Egg	Meat is cooked fried and eaten with Naan Eggs are boiled fried and eaten.	N	Y	N	N	Gujjar, Pahari, Bakarwal	0.22	214
178	*Pucrasia macrolopha* G.R. Gray, 1841 (Phasianidae)	Vankukur (KAS) Jangli Murga (KAS)	Bush meat Gizzard	Both meat and gizzard are cooked and eaten with rice. Soup is made from the meat.	Y	N	N	N	Kashmiri, Gujjar, Pahari, Bakarwal	0.23	219
179	*Streptopelia decaocto* Frivaldszky, 1838 (Columbidae)	Kukil (KAS, (AJK)	Bush meat	Cooked and eaten with rice or Naan (bread).	Y	N	N	Y	Kashmiri, Gujjar, Pahari	0.24	225

180	*Streptopelia orientalis* Latham, 1790 (Columbidae)	Wan Kukil (KAS)	Bush meat	Cooked and eaten with rice or Naan (bread)..	Y	N	N	N	Kashmiri, Gujjar, Pahari	0.24	227
181	*Streptopelia tranquebarica* Hermann,1804 (Columbidae)	Koggi (AJK)	Bush meat	Cooked, fried and eaten with rice or naan (bread).	N	N	N	Y	Kashmiri, Gujjar, Pahari	0.23	219
182	*Tadorna ferruginea,* Pallas, 1764 (Anatidae)	Surkhab (KAS)	Bush meat Gizzard	Both meat and gizzard are cooked eaten with rice.	Y	N	N	N	Kashmiri	0.25	234
183	*Tetraogallus himalayensis* G.R. Gray, 1843 (Phasianidae)	Ripja (LAD)	Bush meat Gizzard	Both meat and gizzard are cooked and eaten with rice or chapatii (bread). Soup is made from the meat.	N	N	Y	N	Brokpa, Beda, Balti	0.23	216
184	*Tetraogallus tibetanus* Gould, 1854 (Phasianidae)	Ticok (LAD)	Bush meat Gizzard	Both meat and gizzard are cooked and eaten with chapatii (bread). Soup is made from the meat.	N	N	Y	N	Brokpa, Beda, Balti	0.23	219
185	*Columba leuconota* Vigors, 1831 (Columbidae)	Mickron (LAD)	Bush meat Gizzard	Both meat and gizzard are cooked and eaten with chapatii (bread).	N	N	Y	N	Brokpa, Beda, Balti	0.23	218

186	*Perdix hodgsoniae* Hodgson, 1857 (Phasianidae)	Srakpa, Riibya (LAD)	Bush meat Gizzard	Both meat and gizzard are cooked and eaten with chapatii (bread).	N	N	Y	N	Beda, Balti, Brokpa	0.21	200
187	*Anser anser* L., 1758 (Anatidae)	Toskar (LAD)	Bush meat Gizzard	Both meat and gizzard are cooked and eaten with chapatii (bread).	N	N	Y	N	Beda, Balti, Brokpa	0.22	206
188	*Anas platyrhynchos* L., 1758 (Anatidae)	Chuybya (LAD)	Bush meat Gizzard	Meat is cooked, fried eaten with rice. Soup is made from the meat.	N	N	Y	N	Brokpa, Balti	0.21	197
Fish											
189	*Barbus sarana* Hamilton, 1822 (Cyprinidae)	Devgaad (KAS) Machlii (JAM, AJK)	Flesh	Smoked, cooked and eaten with rice or chapatii (bread).	Y	Y	N	Y	Kashmiri, Gujjar, Pahari	0.22	210
190	*Catla catla* F. Hamilton, 1822 (Cyprinidae)	Punjaibgaad (KAS) Machlii (JAM, AJK)	Flesh, Coagulated protein	Flesh is smoked, dried, cooked and eaten with rice or chapatii (bread). Coagulated protein is fried and eaten.	Y	Y	N	Y	Kashmiri, Gujjar, Pahari	0.34	325
191	*Cirrhinus cirrhosus* Bloch, 1795 (Cyprinidae)	Chmakgaad (KAS), Machlii (AJK)	Flesh Coagulated protein	Flesh is smoked, dried, cooked and eaten with rice or chapatii (bread).	Y	N	N	Y	Kashmiri, Gujjar, Pahari	0.26	245

				Coagulated protein is fried and eaten.							
192	*Labeo calbasu* Hamilton, 1822 (Cyprinidae)	Machalyiee, Darvaiogaad (AJK)	Flesh, Coagulated protein	Flesh is smoked, cooked and eaten with rice. Coagulated protein is fried and eaten.	N	N	N	Y	Kashmiri, Gujjar, Pahari	0.23	215
193	*Labeo dero* Hamilton, 1822 (Cyprinidae)	Machlii (JAM), Daryaavgaad (AJK)	Flesh Coagulated protein	Flesh is cooked, fried and eaten. Coagulated protein is fried and consumed.	N	Y	N	Y	Kashmiri, Gujjar, Pahari	0.25	241
194	*Oncorhynchus mykiss* Walbaum, 1792 (Salmonidae)	Kulgaad (KAS), Trout (AJK) Nya (LAD)	Flesh	Fried and consumed.	Y	N	Y	Y	Kashmiri, Gujjar, Pahari, Beda	0.21	200
195	*Puntius ticto* F. Hamilton, 1822 (Cyprinidae)	Sungaad (AJK), Machli (JAM)	Flesh	Fried and consumed.	N	Y	N	Y	Kashmir, Gujjar, Pahari	0.30	285
196	*Salmo trutta fario* L., 1758 (Salmonidae)	Naalgaad (KAS), Trout (JAM, AJK), Nya (LAD)	Flesh	Fried and consumed	Y	Y	Y	Y	Kashmiri, Gujjar Pahari, Beda	0.25	236
197	*Schizopyge niger* Heckel, 1838 (Cyprinidae)	Alegaad, Kashir Gaad (KAS)	Flesh Coagulated protein	Flesh is smoked, dried, cooked and consumed with rice. Coagulated protein is	Y	N	N	N	Kashmiri	0.37	352

				fried and consumed. Dried fishes are consumed in harsh winters.							
198	*Schizothorax esocinus* Heckel, 1838 (Cyprinidae)	Chhurru, Kashir Gaad (KAS)	Flesh Coagulated protein	Flesh is smoked, dried, cooked and consumed with rice. Coagulated protein is fried and eaten. Dried fishes are consumed in harsh winters.	Y	N	N	N	Kashmiri	0.25	241
199	*Schizothorax labiatus* McClelland, 1842 (Cyprinidae)	Chush (KAS) , Nya (LAD)	Flesh Coagulated protein	Flesh is smoked, dried, cooked and consumed with rice. Coagulated protein is fried and eaten. Dried fishes are consumed in harsh winters.	Y	N	Y	N	Kashmiri, Beda Balti	0.21	200
200	*Schizothorax plagiostomus* Heckel, 1838 (Cyprinidae)	Khont (KAS), Kashmiri machlii (AJK)	Flesh Coagulated protein	Flesh is smoked, dried, cooked and consumed with rice. Coagulated protein is fried and eaten.	Y	N	N	Y	Kashmiri, Gujjar, Pahari	0.34	324

				Dried fishes are consumed in harsh winters.							
201	*Triplophysa kashmirensis* Hora, 1922 (Nemacheilidae)	Jhelumgaad (KAS), Machli (AJK)	Flesh	Cooked, dried, and consumed with rice.	Y	N	N	Y	Kashmiri, Gujjar, Pahari	0.33	310
202	*Schizothorax curvifrons* Heckel, 1838 (Cyprinidae)	Satar, Kashir gaad (KAS)	Flesh Coagulated proteins	Flesh is smoked, cooked, and eaten with rice. Coagulated protein is fried and consumed.	Y	N	N	N	Kashmiri	0.36	342
203	*Labeo dyocheilus,* McClelland, 1839 (Cyprinidae)	Punjaibgaad (KAS)	Flesh Coagulated protein	Cooked and consumed with rice.	Y	N	N	N	Kashmiri	0.30	285
204	*Crossocheilus latius* Hamilton, 1822 (Cyprinidae)	Naalgaad (KAS), Machlii (JAM)	Flesh	Cooked and consumed with rice.	Y	Y	N	N	Kashmiri	0.29	274
205	*Glyptothorax kashmiriensis* Hora, 1923 (Sisoridae)	Devgaad (KAS) Machi (AJK)	Flesh	Cooked and consumed with rice.	Y	N	N	Y	Kashmiri, Pahari	0.26	246
206	*Crossocheilus diplochilus* Heckel, 1838 (Cyprinidae)	Tetther (KAS)	Flesh	Cooked and consumed with rice.	Y	N	N	N	Kashmiri, Gujjar	0.28	271
207	*Puntius conchonicus* F. Hamilton, 1822 (Cyprinidae)	Roasy (KAS)	Flesh	Cooked and consumed with rice.	Y	N	N	N	Kashmiri	0.25	238
208	*Gambusia affinis* Poey ,1854 (Poeciliidae)	Gambezi (KAS) (JAM)	Flesh	Cooked and consumed with rice.	Y	Y	N	N	Kashmiri	0.22	208

209	*Gambusia alvarezi* Poey ,1854 (Poeciliidae)	Ledargaad (KAS) Machli (JAM)	Flesh	Cooked and consumed with rice.	Y	Y	N	N	Kashmiri	0.25	238

Regions from which species are reported are abbreviated as: Azad Kashmir (AJK), Ladakh (LAD), Jammu (JAM), Kashmir (KAS)
